# Two New Triterpenes from Basidiomata of the Medicinal and Edible Mushroom, *Laetiporus sulphureus*

**DOI:** 10.3390/molecules26237090

**Published:** 2021-11-24

**Authors:** Khadija Hassan, Blondelle Matio Kemkuignou, Marc Stadler

**Affiliations:** 1 Department of Microbial Drugs, Helmholtz Centre for Infection Research and German Centre for Infection Research (DZIF), Partner Site Hannover/Braunschweig, Inhoffenstrasse 7, 38124 Braunschweig, Germany; Khadija.Hassan@helmholtz-hzi.de (K.H.); Blondelle.MatioKemkuignou@helmholtz-hzi.de (B.M.K.); 2 Institute of Microbiology, Technische Universität Braunschweig, Spielmannstraße 7, 38106 Braunschweig, Germany

**Keywords:** *Laetiporus* sp., triterpenoids, antimicrobial, antiproliferative

## Abstract

In the search for novel anti-infectives from natural sources, fungi, in particular basidiomycetes, have proven to still harbor so much potential in terms of secondary metabolites diversity. There have been numerous reports on isolating numerous secondary metabolites from genus *Laetiporus*. This study reports on two new triterpenoids, laetiporins C and D, and four known triterpenes from the fruiting body of *L*. *sulphureus*. The structures of the isolated compounds were elucidated based on their 1D and 2D nuclear magnetic resonance (NMR) spectroscopic data in combination with high-resolution electrospray mass spectrometric (HR-ESIMS) data. Laetiporin C exhibited weak antifungal activity against *Mucor hiemalis.* Furthermore, the compounds showed weak antiproliferative activity against the mouse fibroblast L929 and human cancer cell lines, including KB-3-1, A431, MCF-7, PC-3 and A549.

## 1. Introduction

Fungi are a valuable group of organisms that have come under a role of study due to their potential for biological activity [1]. Mushroom-forming fungi (mostly belonging to the phylum Basidiomycota) are known to be prolific producers of bioactive secondary metabolites with economic importance [2,3]. Some of these metabolites have potential benefits with emphasis on their anti-Alzheimer, antidiabetic, anti-malarial, anti-microbial, anti-oxidant, antitumor, anti-viral and hypocholesterolemic activities which are important medicinal targets in terms of drug discovery today [3,4,5].

The genus *Laetiporus* belongs to the Fomitopsidaceae (Polyporales) [6] and its species form conspicuous basidiomes on wood. *Laetiporus* spp. are considered to be forest pathogens, but some taxa are reported to be edible or to contain medicinal valuable compounds [7,8,9]. The genus is geographically distributed world-wide, and its species can be found in cold temperate to tropical zones. They are most often associated with angiosperm hosts belonging to the Fabaceace, Meliaceae, and Salicaceae families [10]. There are 15 species that have been accepted in the genus worldwide and 11 species have been confirmed in the *L*. *sulphureus* complex by phylogenetic analyses [11].

In the present study, *Laetiporus sulphureus*, a fungus whose fruiting bodies commonly occur in Germany, was investigated. This species is also known under its trivial names such as ‘Chicken-of-the-Woods’ or ‘Chicken Mushroom’ [12,13]. It has been used traditionally as food and for its medicinal properties. In recent years, there have been extensive examinations and research into the biologically active compounds and extracts from the species from this genus and their benefits on human health [12,13]. We have recently come across fresh specimens from German and found interesting metabolite patterns in its fruitbody extracts by HPLC analysis. Hence, we decided to isolate and identified the respective metabolites. This study reports the isolation of two new compounds, for which we propose the trivial names laetiporins C and D, as well as their structure elucidation and biological activities.

## 2. Results and Discussion

### 2.1. Structure Elucidation of Compound **1** and **2**

**Figure 1:**
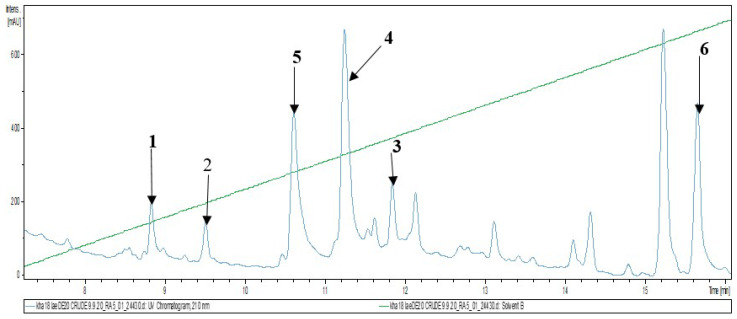
LC–UV/Vis chromatogram of the crude extract from the fruiting body (diode array detection at 200–640 nm). Stationary phase: C18 Acquity UPLC BEH column; for gradient and other details on the experimental setup, see the Experimental section; 1–6: Major metabolites detected (chemical structures see Figure 2) The green diagonal line indicates the gradient (% of acetonitrile).

**Figure 2:**
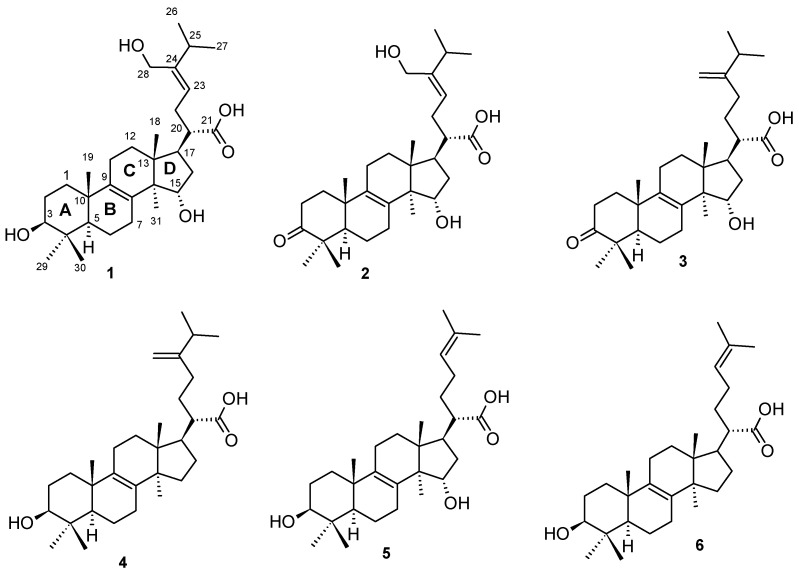
Chemical structures of compounds **1**–**6** isolated from *Laetiporus sulphureus*.

Investigation of the chemical composition of the fruiting body of *Laetiporus sulphureus* and subsequent fractionation and purification by reverse phase HPLC (Figure 1) led to the isolation of two previously undescribed lanostane triterpenoids for which the trivial names laetiporins C (**1**) and D (**2**) were assigned. In addition, four known compounds: fomefficinic acid (**3**) [14], eburicoic acid (**4**) [15], 15 α-hydroxytrametenolic acid (**5**) [16] and trametenolic acid (**6**) [17] were also isolated from the same crude extract (Figure 2). The above-mentioned known compounds except compound **3** were previously isolated from the liquid culture of a Kenyan *Laetiporus* sp. [12]. The structures of the known compounds were elucidated by comparison of their NMR spectroscopic data and HR-ESIMS with those reported in the literature.

Compound **1** was isolated as a dark brown solid with the molecular formula C_31_H_50_O_5_ and 7 degrees of unsaturation established from HR-ESIMS data (positive mode), which displayed molecular ion peaks [M+H-H_2_O]^+^ at *m/z* 485.3628 and [M+Na]^+^ at *m/z* 525.3552. The ^1^H-NMR spectrum exhibited signals of five methyl groups singlet at *δ* 0.70 (H-30, s), *δ* 0.70 (H-18, s), *δ* 0.81 (H-31, s), *δ* 0.90 (H-29, s), *δ* 0.90 (H-19, s), two methyl groups doublet at *δ* 0.94 (H-26/H-27, d, *J* = 6.8), two hydroxymethine at *δ* 3.00 (H-3, td, *J* = 6.02, 10.76) and *δ* 4.01 (H-15, br dd, *J* = 5.81, 10.54), two diasteriotopic protons corresponding to one oxymethylene at *δ* 3.98 (H-28a, br d, *J* = 12.1) and *δ* 3.85 (H-28b, br d, *J* = 12.2), one olefinic protons at *δ* 5.09 (H-23, t, *J* = 7.31) and two hydroxy groups doublet at *δ* 4.30 (OH-3/OH-15, d, *J* = 5.3). The ^13^C NMR spectroscopic data revealed the presence of 31 carbons signals further identified based on combined analysis of 1D and ^1^H-^13^C HSQC spectra as seven methyl groups, eight methylenes, one oxymethylene, four methines, two hydroxymethines, one carboxylic carbon, one olefinic carbon, three non-protonated C-sp^2^ hybridized carbons and four quaternary C-sp^3^ hybridized carbons (Table 1).

**Figure 3:**
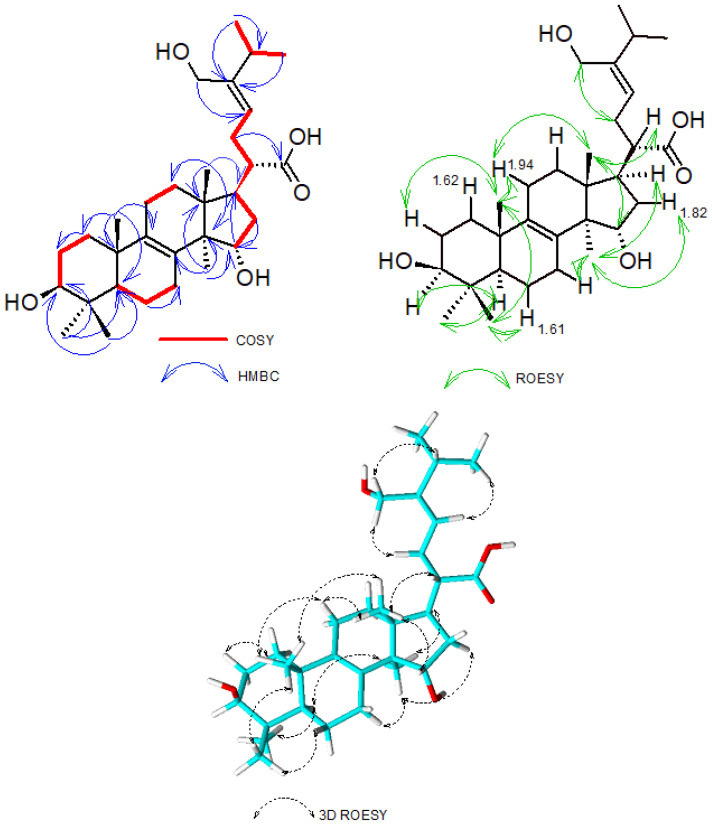
HMBC, COSY, and ROESY correlations of compound **1**.

Analysis of its HMBC spectrum showed correlations of H-18 (*δ* 0.70) to C-12 (*δ* 29.0)/C-13 (*δ* 44.1)/C-14 (*δ* 50.8)/C-17 (*δ* 45.3), H-19 (*δ* 0.90) to C-1 (*δ* 35.2)/C-5 (*δ* 50.0)/C-9 (*δ* 133.9)/C-10 (*δ* 36.6), H-29 (*δ* 0.90) and H-30 (*δ* 0.70) to C-3 (*δ* 76.7) /C-4 (*δ* 38.5)/C-5 (*δ* 50.0) and H-31 (*δ* 0.81) to C-13 (*δ* 44.1)/C-14 (*δ* 50.8)/C-15 (*δ* 71.0) which suggested the presence of a lanostane skeleton as previously reported by [12]. The position of the double bond ∆^8–9^ was confirmed on the HMBC spectrum where correlations between H-31 (*δ* 0.81) and C-8 (*δ* 133.9) and between H-19 (*δ* 0.90) and C-9 (*δ* 134.0) was observed. The side chain connection was established from the HMBC correlation of H-20 (*δ* 2.02) to C-17 (*δ* 45.3). Further ^3^J-HMBC correlation between the methylene group H-22 (*δ* 2.15) and C-21 (*δ* 176.8) confirmed the position of the carboxylic group connected at C-20. Moreover, combined analysis of HMBC, COSY and ROESY spectroscopic data of compound **1** confirmed the positioning and assignment of protons and carbons of the side chains (Figure 3). Most particularly, the HMBC correlations observed between H-28a/H-28b and C-23/C-24/C-25 allowed the positioning of the oxymethylene C-28 placed at C-24. The Z-configuration of the ∆^23–24^ double bond was established based on ROESY correlations observed between H-28a/H-28b and H-22 and between H-23 and H-25. In the COSY spectrum, correlations of H-20 to H-17/H-22, H-22 to H-23, H-25 to H-26/H-27 were observed. Further COSY correlations between H-1 and H-2, H-3 and H-2, H-6 and H-5/H-7, H-11 and H-12, H-14 and H-15 were also recorded. A network ROESY correlations, which gave an indication of the orientation in space of some groups at various stereocenters were observed and further allowed the establishment of the absolute configuration of compound **1**. The α-orientation of H-3 was suggested based on the ROESY correlations of H-3 to H-5/H-29, as well as the coupling constant *J* = 5.30 and 10.54 Hz observed for this proton. Therefore, the S configuration was assigned at C-3 as previously reported by [12]. By using the orientation of H-3 and the S configuration assigned to C-3 as reference, the configuration of the other stereocenters was determined based on ROESY correlations observed between H-5 to H-29, H-30 to H_β_-6 (1.61)/H-19, H-19 to H_β_-11, H-18 to H_β_-11 (1.94)/H-19/H-20/H-15, H-31 to H_α_-16 (1.82)/H-17. These correlations allowed the assignment of the absolute configuration of compound **1** as 3*S*, 5*R*, 10*S*, 13*R*, 14*R*, 15*S*, 17*R*, 20*S* and the trivial name laetiporin C was attributed to it.

Compound **2** was obtained as a dark brown solid. The molecular formula of C_31_H_48_O_5_ (eight degrees of unsaturation) were deduced from the HR-ESIMS data (positive mode) where molecular ion peaks [M+H-H_2_O]^+^ at *m/z* 483.3466, [M+H]^+^ at *m/z* 501.3572 and [M+Na]^+^ at *m/z* 523.3394 were observed. The molecular formula assigned to compound **2** showed a deficiency of two hydrogens compared to compound **1** suggesting that an oxidation occurred. Extensive analysis of the 1D and 2D-NMR spectroscopic data of **2** indicated its close similarity with compound **1** with the only difference being the presence in its structure of a keto carbonyl group at position C-3 instead of the hydroxyl group as in **1**. This was confirmed on its ^1^H-NMR and ^13^C-NMR spectra, where the oxygenated methine signal resonating at *δ* 3.00 (H-3, br dd, *J* = 5.3, 10.54) and *δ* 76.74 (C-3) in compound **1** was missing and instead a keto carbonyl group was observed at *δ* 216.3. HMBC correlations of diasteriotopic protons H_α_-1 (δ 1.52), H_β_ -1 (δ 1.89), H_α_-2 (δ 2.52) and H_β_ -2 (δ 2.33) as well as correlations of H-29 (δ 1.00), H-30 (δ 0.96) to carbon C-3 (*δ* 216.3) further supported the assignment of the structure of compound **2**, for which we propose the trivial name laetiporin D.

### 2.2. Physico-Chemical Characteristics of Compounds **1** and **2**

Laetiporin C (**1**): Dark brown solid. [α]^20^_D_ = +85°, UV (DMSO) λmax (log ε) 256 (3.69) HR-ESIMS *m/z* 485.3628 [M+H-H_2_O]^+^, *m/z* 525.3552 [M+Na]^+^ (calcd. for C_31_H_50_NaO_5_, 525.3550). For NMR data, see Table 1.

Laetiporin D (**2**): Dark brown solid. [α]^20^_D_ = +62.8°, UV (DMSO) λmax (log ε) 256 (3.67) HR-ESIMS *m/z* 483.3466 [M+H-H_2_O]^+^, *m/z* 501.3572 [M+H]^+^, *m/z* 523.3394 [M+Na]^+^ (calcd. for C_31_H_48_NaO_5_, 523.3394). For NMR data, see Table 1.

### 2.3. Biological Assays

The new lanostanoids laetiporins C (**1**) and D **(2**) were subjected to both, antimicrobial and cytotoxicity assays. For the antimicrobial assay with a wide array of organisms, only laetiporin C showed weak inhibitory effect against *Mucor hiemalis* (DSM 2656) with MIC 66 µg/mL. On the other hand, no inhibition was observed against the other test organisms with both compounds. The isolation of triterpenoids from both edible and inedible mushrooms and their activities has been widely documented in recent years [12,18]. Compounds from mushrooms, for instance, are known to have antimicrobial activity [19].

On the other hand, despite there being numerous reports on the biological activities of the natural lanostane-type triterpenoids like inhibit tumor cell growth, induce apoptosis, and inhibit angiogenesis and metastasis [20,21], these compounds did not have cytotoxic activity in all 6 cell lines. However, there was cell growth inhibition illustrated by five cell lines including L929, A431, A549, PC-3, and MCF-7 with later showing most cell growth inhibited by both compounds. Compound **3** was inactive and the other known metabolites **4**–**6** were not tested, as their activities have previously been reported [12]. A detailed report on their activity is given in the SI (Table S3).

## 3. Materials and Methods

### 3.1. General Information

HPLC-DAD/MS measurements were performed using an amaZon speed ETD (electron transfer dissociation) ion trap mass spectrometer (Bruker Daltonics, Bremen, Germany) and measured in positive and negative ion modes simultaneously. HPLC system (column C18 Acquity UPLC BEH (Waters, Milford, MA USA), solvent A: H_2_O; solvent B: acetonitrile (ACN) supplemented with 0.1% formic acid, gradient conditions: 5% B for 0.5 min, increasing to 100% B in 20 min, maintaining isocratic conditions at 100% B for 10 min, flow rate 0.6 mL/min, UV/Vis detection 200–600 nm).

HR-MS (high-resolution mass spectrometry) data were recorded on a MaXis ESI-TOF (electrospray ionization-time of flight) mass spectrometer (Bruker Daltonics) coupled to an Agilent (Santa Clara, CA, USA) 1260 series HPLC-UV system and equipped with C18 Acquity UPLC BEH (ultraperformance liquid chromatography) (ethylene bridged hybrid) (Waters) column; DAD-UV detection at 200–600 nm; solvent A (H_2_O) and solvent B (ACN) supplemented with 0.1% formic acid as a modifier; flowrate 0.6 mL/min, 40 °C, gradient elution system with the initial condition 5% B for 0.5 min, increasing to 100% B in 19.5 min and holding at 100% B for 5 min. To determine the molecular formula, Compass DataAnalysis 4.4 SR1 was used using the Smart Formula algorithm (Bruker Daltonics).

NMR spectra were collected on a Bruker 700 MHz Avance III spectrometer equipped with a 5 mm TCI cryoprobe (1H: 700 MHz, 13C: 175 MHz), locked to the respective deuterium signal of the solvent. Optical rotations were measured using Anton Paar MCP-150 Polarimeter (Graz, Austria) with 100 mm path length and sodium D line at 589 nm. The UV spectra were measured on a Shimadzu (Kyoto, Japan) UV/Vis 2450 spectrophotometer using DMSO (Uvasol, Merck, Darmstadt, Germany) as a solvent.

### 3.2. Fungal Material

*Laetiporus sulphureus* was collected in Braunschweig-Riddagshausen, nature reserve. Plane-table sheet 3729 (1:25,000), 80 m NHN. Hosts plants were *Salix spec*, *Fagus sylvatica*, and *Robinia pseudoacacia*. The species, which is the only one of the genus known from Central Europe, was identified by Harry Andersson based on morphological data and the identify was confirmed by one of the authors (M.S). A voucher specimen is deposited in the fungarium of the HZI, Braunschweig.

### 3.3. Extraction of the Crude Extract

The fresh specimen (in total 0.8 kg) were submerged in 4L acetone crushed by a homogenizer and left overnight before extraction [22]. The material was then put in ultrasonic bath for 30 min before it was filtered, and the solvent evaporated. The remaining aqueous phase (50 mL) was suspended in an equal amount of distilled water then added to an equal amount (100 mL) of ethyl acetate, which was later separated by filtration. The supernatant was extracted and filtered through anhydrous sodium sulphate. The resulting ethyl acetate extract was evaporated to dryness by means of rotary evaporator yielding a yellow solid 2.1 g of crude extract.

### 3.4. Isolation of Compounds

The crude extract was fractionated using preparative reverse phase liquid chromatography (PLC 2020, Gilson, Middleton, WI, USA). VP Nucleodur 100-5C 18 ec column (250 × 40 mm, 7 μm: Macherey-Nagel) used as stationary phase. Deionized water (Milli-Q, Millipore, Schwalbach, Germany) (solvent A) and acetonitrile (solvent B) with 0.05% TFA was used as eluent with rate of 40 mL/min. The elution gradient used was 5–100% solvent B in 60 min and thereafter isocratic condition at 100% solvent B for 5 min. UV detection was carried out at 210–600 nm. Nine fractions were collected according to the observed peaks.

Out of those, Fractions F3–F5 were combined and further subjected to preparative HPLC with an elution gradient 20–80% solvent B for 40 min 80–100% for 5 min and finally isocratic condition at 100% solvent B for 5 min. Five fractions A1-A5 were collected from this experiment. Fraction A3 was further purified with elution gradient of 40−60% solvent B in 35 min to give compound **2** (4.8 mg). A similar gradient was applied to fraction A2 to give compound **1** (2.3 mg). Fraction A4 was also purified by reverse phase LC (solvent A/solvent B), elution gradient 45−70% solvent B for 47 min, followed by a gradient shift from 70% to 100% in 5 min, and finally isocratic condition at 100% solvent B for 5 min to afford 1.21 mg of compound **3,** compound **4** (2.66 mg) and compound **5** (2.38 mg). Compound **6** (4.57 mg), was obtained from F6 with an elution gradient 75−90% solvent B for 25 min, followed by a gradient shift from 90% to 100% in 5 min, and finally isocratic condition at 100% solvent B for 5 min.

### 3.5. Cytotoxicity Assay

A panel of six mammalian cell lines including mouse fibroblast L929, HeLa (KB-3-1), epidermoid carcinoma cells A431, breast cancer cells MCF-7, prostate cancer cells PC-3 and adenocarcinomic human alveolar basal epithelial cells A549 were chosen in this assay to determine in vitro cytotoxicity (IC_50_) of compounds **1** and **2**. The cell lines were purchased from the DSMZ collection (Braunschweig, Germany). The cell lines L929, A549 and KB-3-1 were cultured in Dulbecco’s modified Eagle’s medium (DMEM; Gibco; Termo Fisher Scientific, Dreieich, Germany); MCF-7 and A431 cells were cultured in RPMI-1640 medium (Gibco) and PC-3 cells in F12K (Gibco) medium. All media were supplemented with 10% fetal bovine serum (FBS; Gibco) and incubated under 5% CO_2_ at 37 °C for 5 days. The cytotoxicity assay was performed using the MTT (3-(4,5-dimethylthiazol-2-yl)-2, 5 diphenyltetrazolium bromide) test in 96-well microplates in accordance with our standard protocol which have been well previously described [23,24,25]. Methanol was used as negative control, and epothilone B as the positive control. A detailed protocol and parameters used is given in the Supplementary Materials.

### 3.6. Antimicrobial Assay

The minimum inhibition concentrations (MIC) were determined in serial dilution assay against various test organisms including bacteria: *Bacillus subtilis* (DSM 10), *Staphylococcus aureus* (DSM 346), *Acinetobacter baumanii* (DSM 30008), *Chromobacterium violaceum* (DSM 30191), *Escherichia coli* (DSM 1116), *Pseudomonas aeruginosa* (PA14); *Mycobacteria*: *Mycolicibacterium smegmatis* (ATCC 700084); Fungi: *Candida albicans* (DSM1665), *Schizosaccharomyces pombe* (DSM 70572), *Mucor hiemalis* (DSM 2656), *Pichia anomala* (DSM 6766), and *Rhodotorula glutinis* (DSM 10134). The assay was excecuted in accordance with literature descriptions [25] and a detailed protocol can be found in the Supplementary Materials.

## 4. Conclusions

Two new triterpenes from *L*. *sulphureus* fruiting body, denoted as laetiporins C and D (**1** and **2**), were isolated in this study. In addition, four known triterpenoids, fomefficinic acid, eburicoic acid, 15 α-hydroxytrametenolic acid and trametenolic acid (**3**–**6**) were also isolated and characterized. The latter compounds except for fomeffinic acid were already isolated previously by Chepkirui et al. [12] from cultures of an African *Laetiporus* sp. but found devoid of antimicrobial effects and had weak cytotoxicity. Several other triterpenes like laetiposides A–D, laetiporins A–B, sulphurenic acid and dehydroeburicoic acid just to mention these few, have also been isolated previously from *Laetiporus* spp. This genus could therefore be considered as a prolific source for isolation of lanostanoids-like triterpenes. Although the triterpenes isolated exhibited no cytotoxic effect against the cancer cell lines, the compounds showed antiproliferative activity against the cancer cell lines tested. Laetiporin C (**1**) only showed weak antifungal activity against the sensitive non-pathogenic zygomycete *Mucor hiemalis*. Even though the observed activities were relatively weak, the isolated compounds are now available for the first time to be tested as pure compounds in a library of fungal metabolites and can be tested for further biological activities.

## Figures and Tables

**Table 1:** ^13^C and ^1^H-NMR spectroscopic data (^1^H 700 MH_Z_, ^13^C 175 MH_Z_ in DMSO-*d*6, *δ* in ppm) for compounds **1** and **2**.

Pos.	1		2	
	*δ*_C,_ Type	*δ*_H_ (*J* in Hz)	*δ*_C,_ Type	*δ*_H_ (*J* in Hz)
1	35.2, CH_2_	α:1.10 (m) ^a^ β:1.62 (m) ^a^	35.4, CH_2_	α: 1.52 (m) ^a^ β: 1.89 (m) ^a^
2	27.6, CH_2_	1.48 (m) ^a^	34.0, CH_2_	α: 2.52(m) ^b^ β: 2.33 (m) ^a^
3	76.7, CH	3.00 (br dd), *J* = 5.30, 10.54	216.3, C	-
4	38.5, C	-	46.6, C	-
5	50.0, CH	0.92 (m) ^a^	50.5, CH	1.50, m
6	17.9, CH_2_	α:1.41 (m) ^a^ β:1.61 (m) ^a^	19.0, CH_2_	α:1.50 (m) ^a^ β:1.58 (m) ^a^
7	26.5, CH_2_	2.11 (m)^a^	26.4, CH_2_	2.15 (m) ^a^
8	133.9, C	-	134.7, C	-
9	134.0, C	-	132.7, C	-
10	36.6, C	-	36.5, C	-
11	20.1, CH_2_	α: 1.87 (m) ^a^ β: 1.94 (m) ^a^	20.2, CH_2_	1.94 (m) ^a^
12	29.0, CH_2_	α:1.29 (m) ^a^ β:1.64 (m) ^a^	29.1, CH_2_	α:1.28 (m) ^a^ β:1.64 (m) ^a^
13	44.1, C	-	44.1, C	-
14	50.8, C	-	50.9, C	-
15	71.0, CH	4.01, (dd), *J* = 10.54, 5.81	71.0, CH	4.04, (br dd), *J* = 10.53, 5.80
16	37.7, CH_2_	α: 1.82 (m) ^a^ β: 1.65 (m) ^a^	37.7, CH_2_	α: 1.84 (m) ^a^ β: 1.64(m) ^a^
17	45.3, CH	2.01 (m) ^a^	45.3, CH	2.03 (m) ^a^
18	15.8, CH_3_	0.70 (s)	16.2, CH_3_	0.74 (s)
19	18.9, CH_3_	0.90 (s)	18.3, CH_3_	1.02 (s)
20	48.1, CH	2.02 (m) ^a^	48.0, CH	2.04 (m) ^a^
21	176.8,C	-	176.8,C	-
22	29.9, CH	2.15 (m) ^a^	29.9, CH	2.15 (m) ^a^
23	120.6, CH	5.09 (t), *J* = 7.31	120.6, CH	5.11 (t), *J* = 7.17
24	146.3, C	-	146.4, C	-
25	31.4, CH	2.36 (sep) *J* = 7.32	31.4, CH	2.36 (m)
26	21.9, CH_3_	0.94 (d), *J* = 6.80	21.9, CH_3_	0.94 (d) *J* = 6.71
27	21.8, CH_3_	0.94 (d), *J* = 6.80	21.8, CH_3_	0.94 (d) J = 6.71
28	57.5, CH_2_	3.98 (br d), *J* = 12.05 3.85 (br d), *J* = 12.21	57.5, CH_2_	3.99 (dd) *J* = 12.21, 5.19 3.86 (dd) *J* = 12.21, 5.34
29	28.1, CH_3_	0.90 (s)	26.1, CH_3_	1.00 (s)
30	15.8, CH_3_	0.70 (s)	20.9, CH_3_	0.96 (s)
31	17.4, CH_3_	0.81 (s)	17.5, CH_3_	0.85 (s)
3-OH	-	4.30 (d) *J* = 5.30	-	-
15-OH	-	4.30 (d) *J* = 5.30	-	4.34 (d) *J* = 5.65

^a^ Signals partially obscured, ^b^ Overlapping with solvent peak.

## Data Availability

Not applicable.

## Supplementary Materials

The following are available online, Figures S1–S37: ESIMS, HR-ESIMS, 1D and 2D NMR spectra of compounds **1**–**6**, Tables S1–S3: Results of biological assays, protocols.

## Sample Availability

Samples of the compounds **1**–**6** are available from the authors.
