# Supplementary material for: Two New Triterpenes from Basidiomata of the Medicinal and Edible Mushroom, Laetiporus sulphureus
Source: Molecules. 2021 Nov 24;26(23):7090. doi: 10.3390/molecules26237090 (PMC8658958; doi:10.3390/molecules26237090)
Supplement: Supplementary file 1 [file molecules-26-07090-s001.zip › molecules-1472587-supplementary.pdf]

Article

Two New Triterpenes from Basidiomata of the Medicinal and Edible Mushroom, *Laetiporus sulphureus*

Khadija Hassan [1,2,†], Blondelle Matio Kemkuignou [1,2,†] and Marc Stadler [1,2,*]

[1] Department of Microbial Drugs, Helmholtz Centre for Infection Research and German Centre for Infection Research (DZIF), Partner Site Hannover/ Braunschweig, Inhoffenstrasse 7, 38124 Braunschweig, Germany; Khadija.Hassan@helmholtz-hzi.de (K.H.); Blondelle.MatioKemkuignou@helmholtz-hzi.de (B.M.K.)

[2] Institute of Microbiology, Technische Universität Braunschweig, Spielmannstraße 7, 38106 Braunschweig, Germany

* Correspondence: Marc.Stadler@helmholtz-hzi.de; Tel.: +49-531-6181-4240

† These authors contributed equally to this work.

Contents

Figure S1: HR-ESIMS data for Laetiporin C (**1**). 4

Figure S2: ESIMS data for Laetiporin C (**1**).......... 5

Figure S3: ^{1}H NMR spectrum (DMSO-d_6, 700 MHz) of Laetiporin C (**1**).......... 6

Figure S4: ^{13}C NMR spectrum (DMSO-d_6, 700 MHz) of Laetiporin C (**1**). 7

Figure S5: HSQC spectrum (DMSO-d_6, 700 MHz) of Laetiporin C (**1**).......... 8

Figure S6: COSY spectrum (DMSO-d_6, 700 MHz) of Laetiporin C (**1**). 9

Figure S7: HMBC spectrum (DMSO-d_6, 700 MHz) of Laetiporin C (**1**).......... 10

Figure S8: ROESY spectrum (DMSO-d_6, 700 MHz) of Laetiporin C (**1**). 11

Figure S9: HR-ESIMS data for Laetiporin D (**2**). 12

Figure S10: ESIMS data for Laetiporin D (**2**) 13

Figure S11: ^{1}H NMR spectrum (DMSO-d_6, 700 MHz) of Laetiporin D (**2**).......... 14

Figure S12: ^{13}C NMR spectrum (DMSO-d_6, 700 MHz) of Laetiporin D (**2**).......... 15

Figure S13: HSQC spectrum (DMSO-d_6, 700 MHz) of Laetiporin D (**2**). 16

Figure S14: COSY spectrum (DMSO-d_6, 700 MHz) of Laetiporin D (**2**). 17

Figure S15: HMBC spectrum (DMSO-d_6, 700 MHz) of Laetiporin D (**2**)....... 18

Figure S16: HMBC spectrum (DMSO-d_6, 700 MHz) of Laetiporin D (**2**)....... 19

Figure S17: ROESY spectrum (DMSO-d_6, 700 MHz) of Laetiporin D (**2**)....... 20

Figure S18: ESIMS data for fomefficinic acid D (**3**)....... 21

Figure S19: ^{1}H NMR spectrum (DMSO-d_6, 700 MHz) of fomefficinic acid D (**3**)....... 22

Figure S20: COSY spectrum (DMSO-d_6, 700 MHz) of fomefficinic acid D (**3**)....... 23

Figure S21: HSQC spectrum (DMSO-d_6, 700 MHz) of fomefficinic acid D (**3**)....... 24

Figure S22: HMBC spectrum (DMSO-d_6, 700 MHz) of fomefficinic acid D (**3**)....... 25

Figure S23:ESIMS data of eburicoic acid (**4**)....... 26

Figure S24: ^{1}H NMR spectrum (DMSO-d_6, 700 MHz) of eburicoic acid (**4**)....... 27

Figure S25: COSY spectrum (DMSO-d6, 700 MHz) of eburicoic acid (**4**)....... 28

Figure S26: HSQC spectrum (DMSO-d_6, 700 MHz) of eburicoic acid (**4**)....... 29

Figure S27: HMBC spectrum (DMSO-d_6, 700 MHz) of eburicoic acid (**4**)....... 30

Figure S28: ESIMS data of 15α-hydroxytrametenolic acid (**5**)....... 31

Figure S29: ^{1}H NMR spectrum (DMSO-d_6, 700 MHz) of 15α-hydroxytrametenolic acid (**5**)....... 32

Figure S30: COSY spectrum (DMSO-d_6, 700 MHz) of 15α-hydroxytrametenolic acid (**5**)....... 33

Figure S31: HSQC spectrum (DMSO-d_6, 700 MHz) of 15α-hydroxytrametenolic acid (**5**)....... 34

Figure S32: HMBC spectrum (DMSO-d_6, 700 MHz) of 15α-hydroxytrametenolic acid (**5**)....... 35

Figure S33: ESIMS data of trametenolic acid (**6**)....... 36

Figure S34: ^{1}H NMR spectrum (DMSO-d_6, 700 MHz) of trametenolic acid (**6**)....... 37

Figure S35: COSY spectrum (DMSO-d_6, 700 MHz) of trametenolic acid (**6**)....... 38

Figure S36: HSQC spectrum (DMSO-d_6, 700 MHz) of trametenolic acid (**6**)....... 39

Figure S37: HMBC spectrum (DMSO-d_6, 700 MHz) of trametenolic acid (**6**)....... 40

Table S1: MIC assay experiment parameters…….41
Antimicrobial assay protocol……..41
Table S2: Cytotoxicity assay experiment parameters……………………………………………………………………………………………..43
Cytotoxicity assay protocol………43
Table S3: Antimicrobial and cytotoxic activities of compounds 1 and 2……………………………………………………………………..44

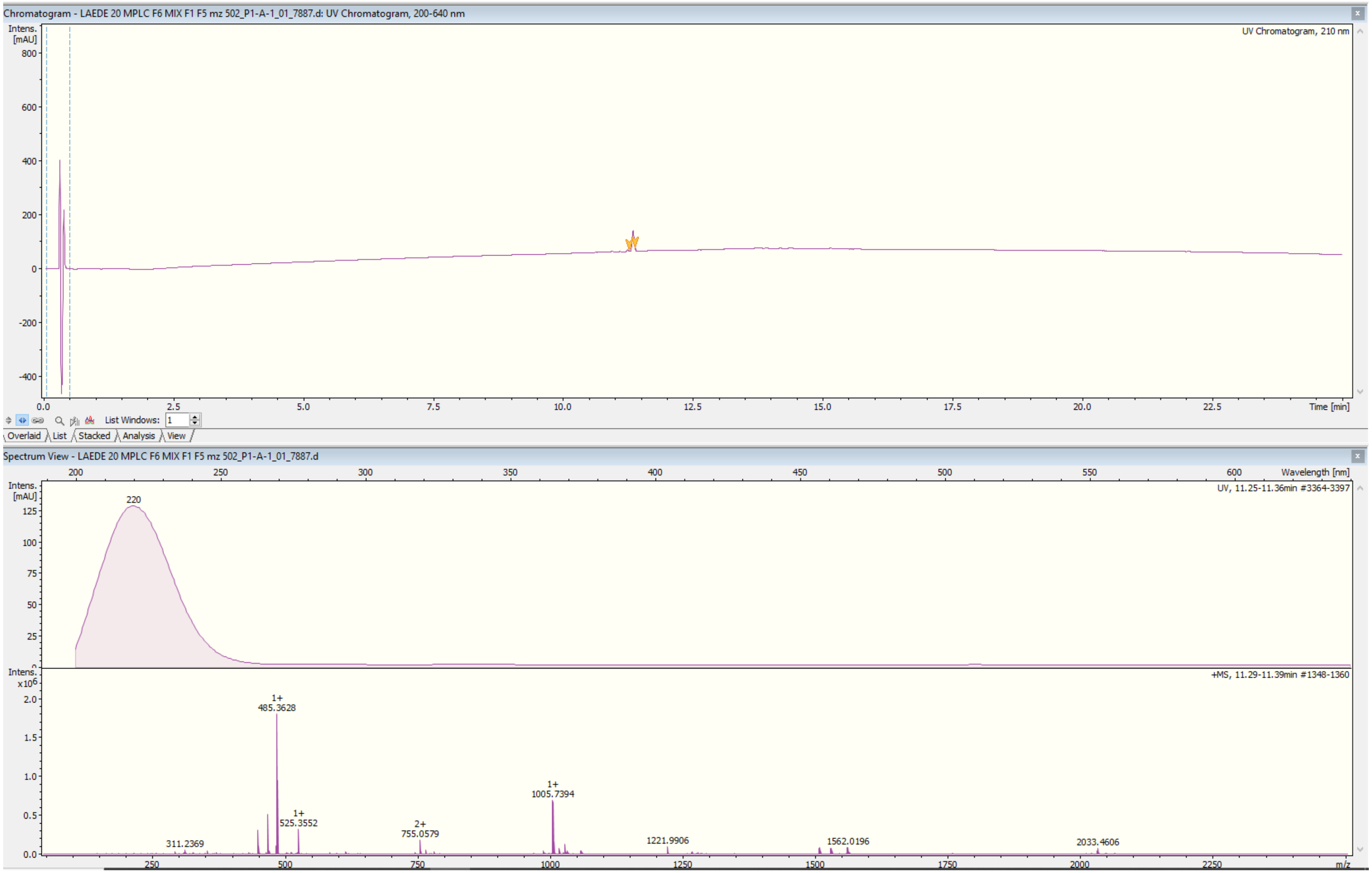

Figure S1: HR-ESIMS data for laetiporin C (**1**).

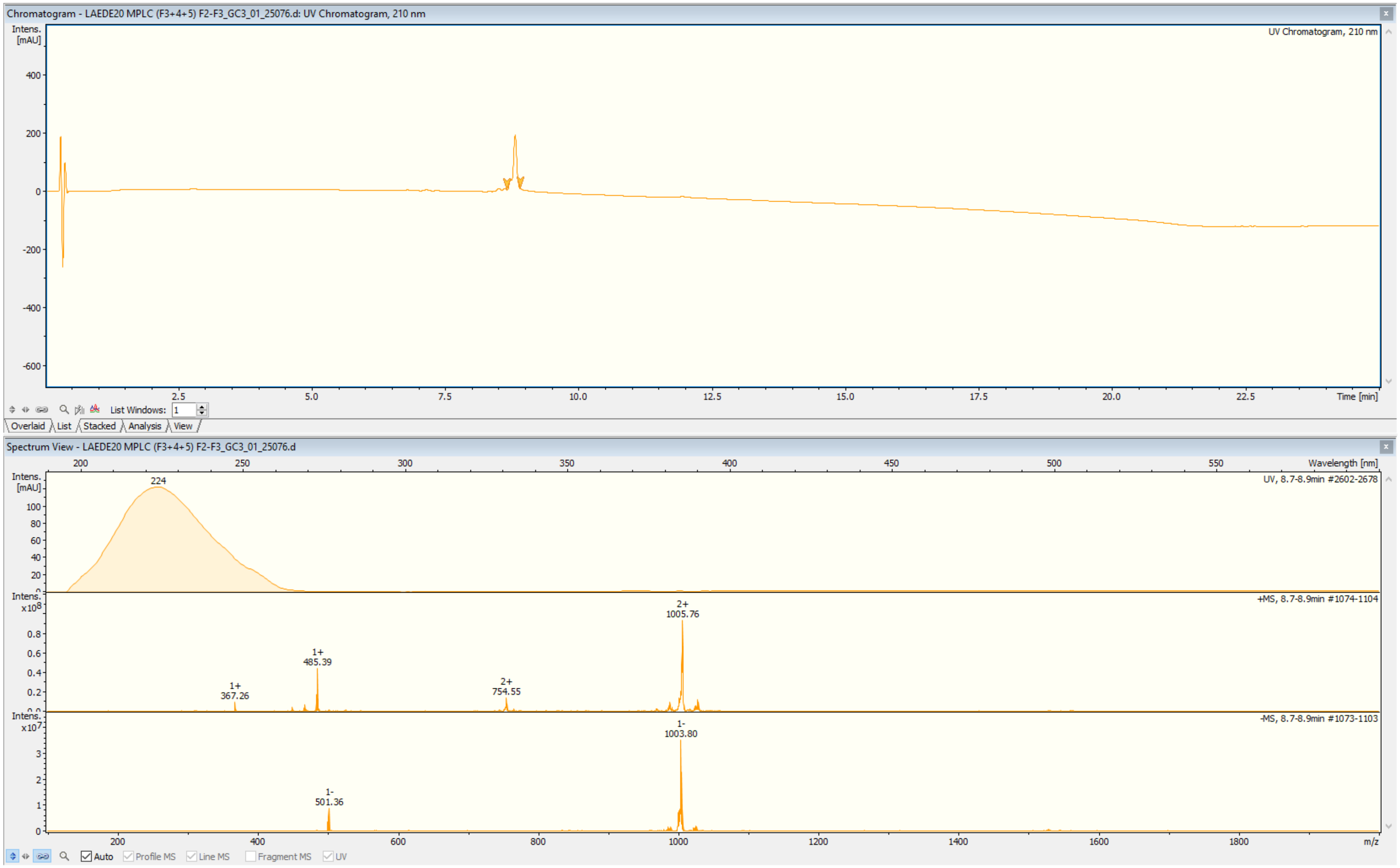

Figure S2: ESIMS data for laetiporin C (**1**)

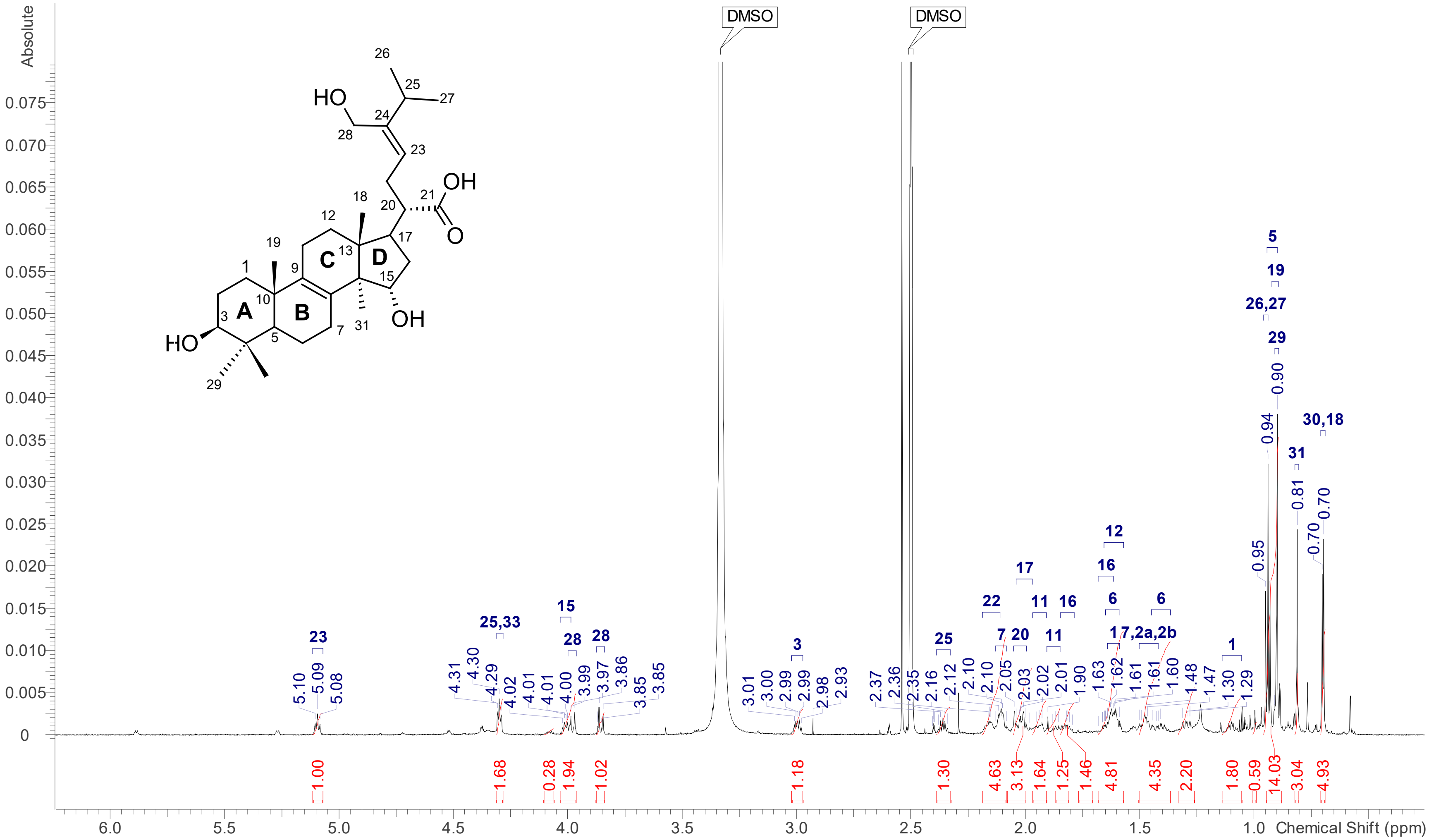

Figure S3: ^{1}H NMR spectrum (DMSO-d_6, 700 MHz) of laetiporin C (**1**).

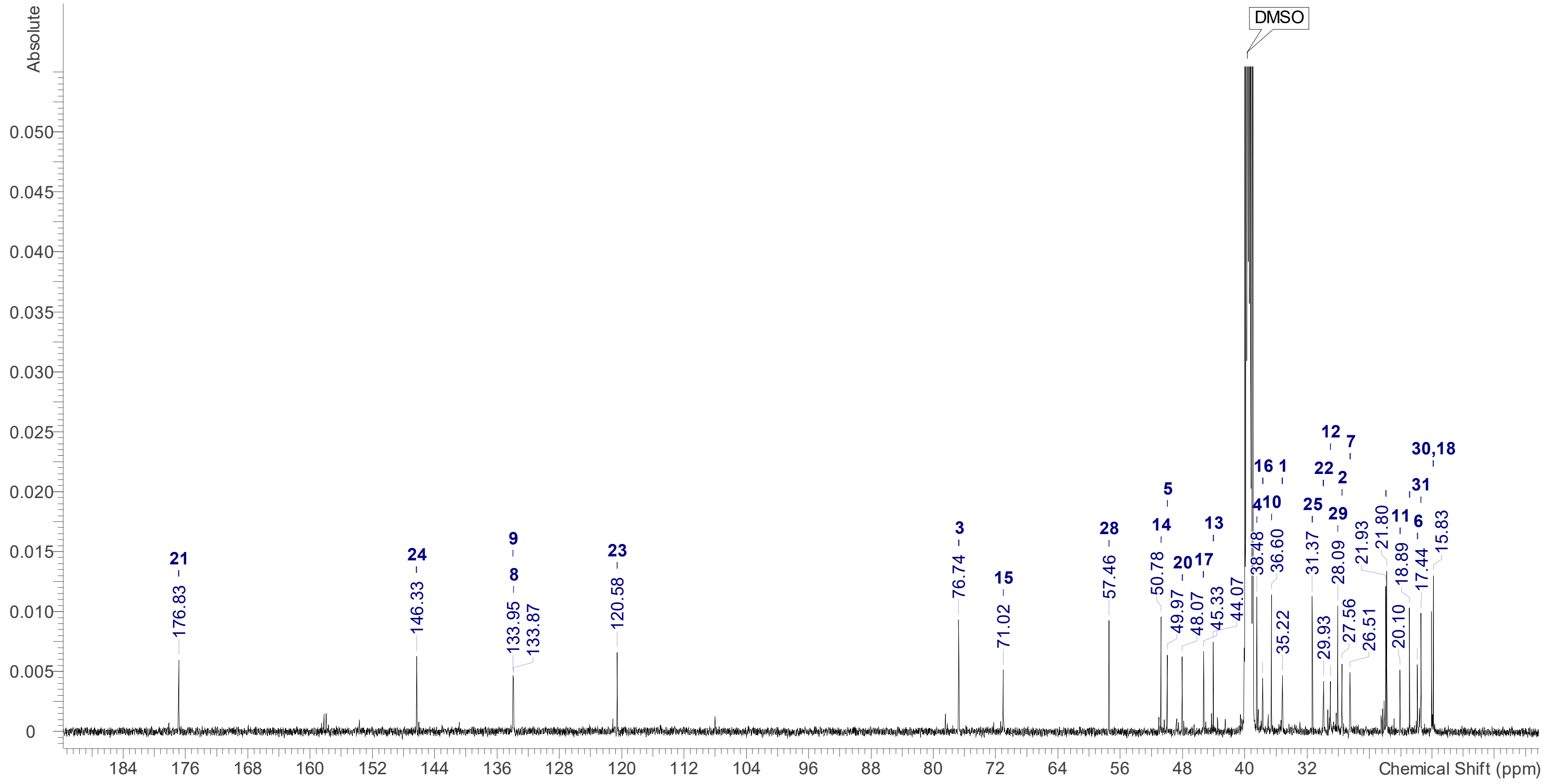

Figure S4: ^{13}C NMR spectrum (DMSO-d_6, 700 MHz) of laetiporin C (**1**).

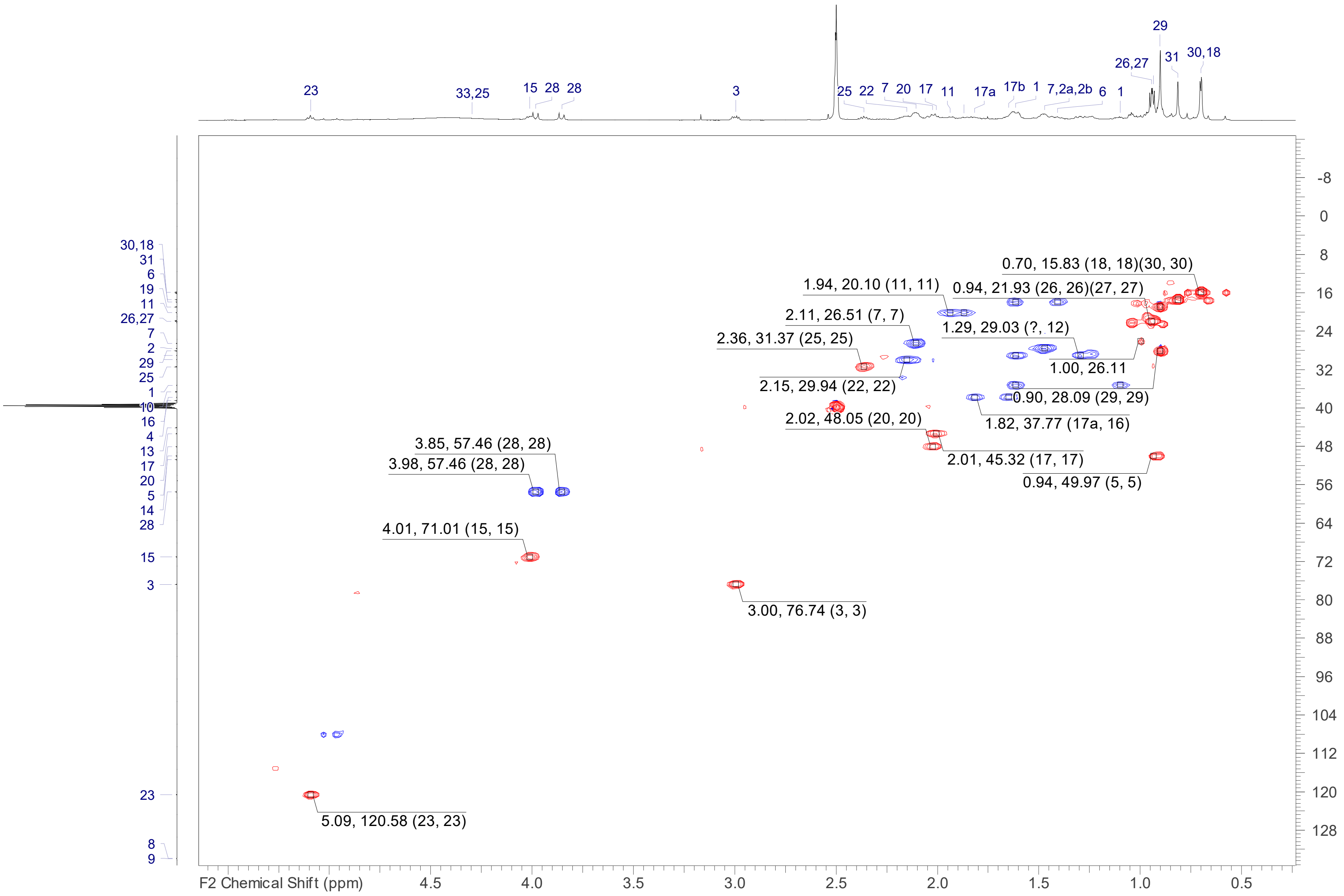

Figure S5: HSQC spectrum (DMSO-d_6, 700 MHz) of laetiporin C (**1**).

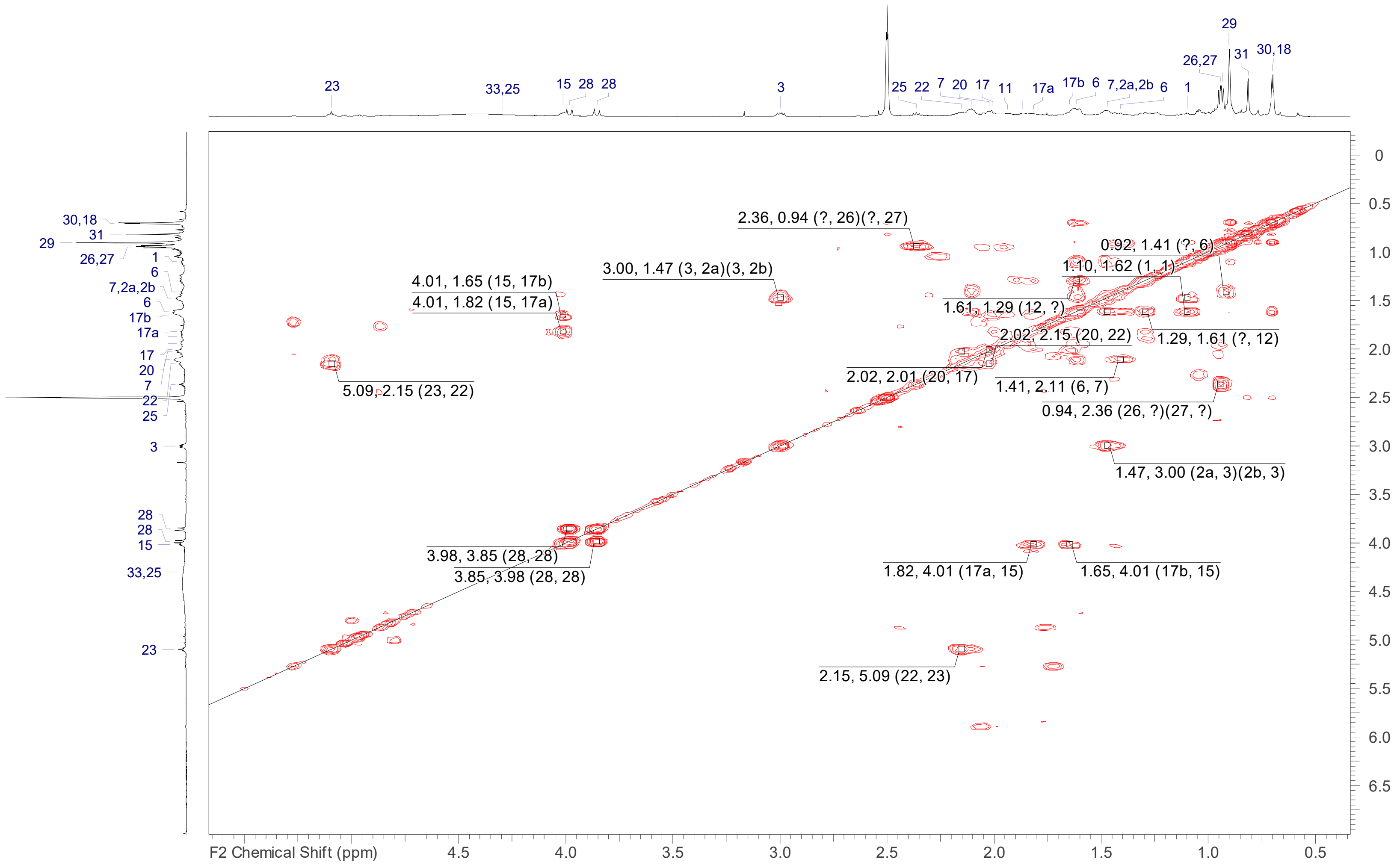

Figure S6: COSY spectrum (DMSO-d_6, 700 MHz) of laetiporin C (**1**).

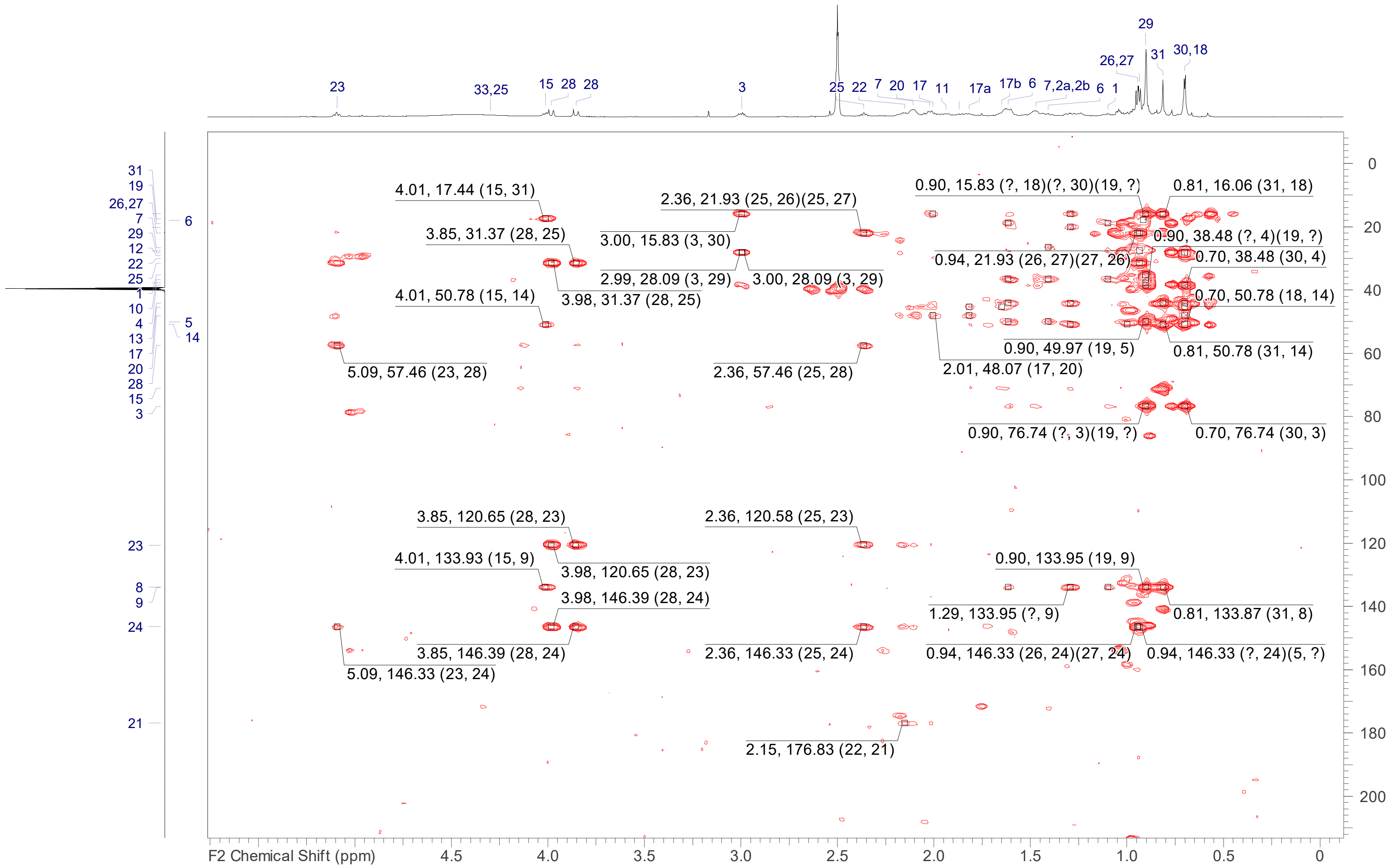

Figure S7: HMBC spectrum (DMSO-d_6, 700 MHz) of laetiporin C (**1**).

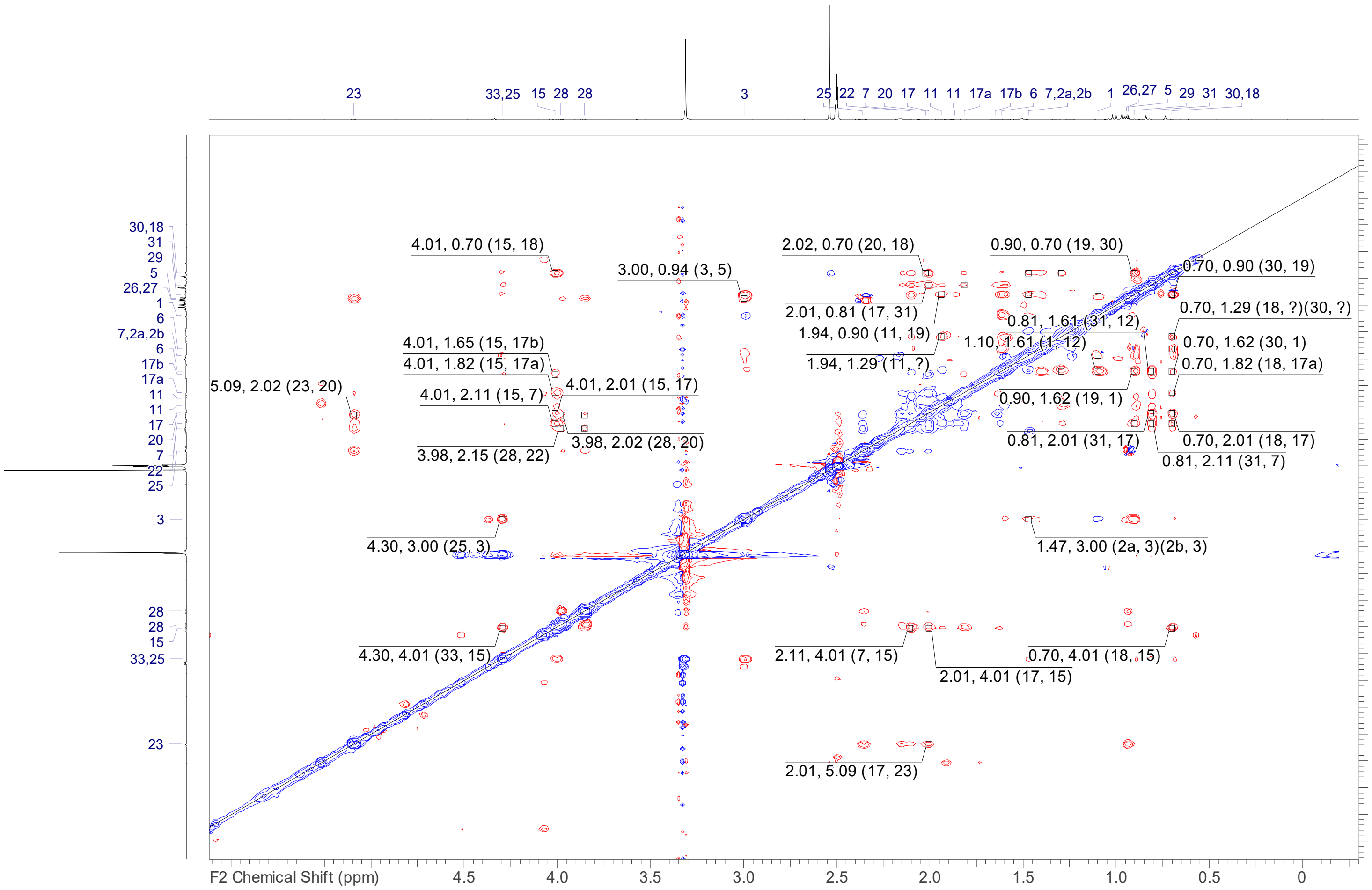

Figure S8: ROESY spectrum (DMSO-d_6, 700 MHz) of laetiporin C (**1**).

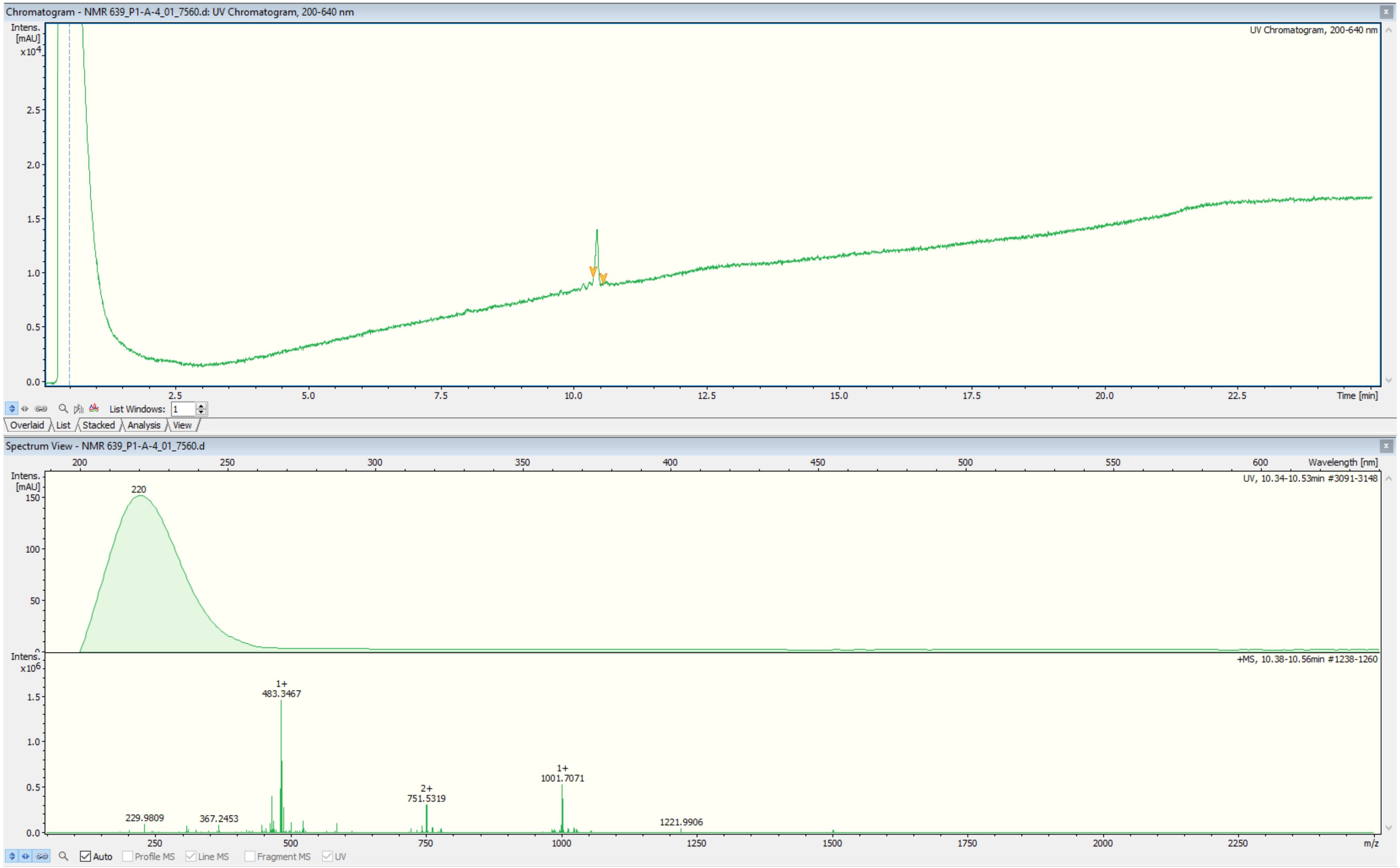

Figure S9: HR-ESIMS data for laetiporin D (**2**).

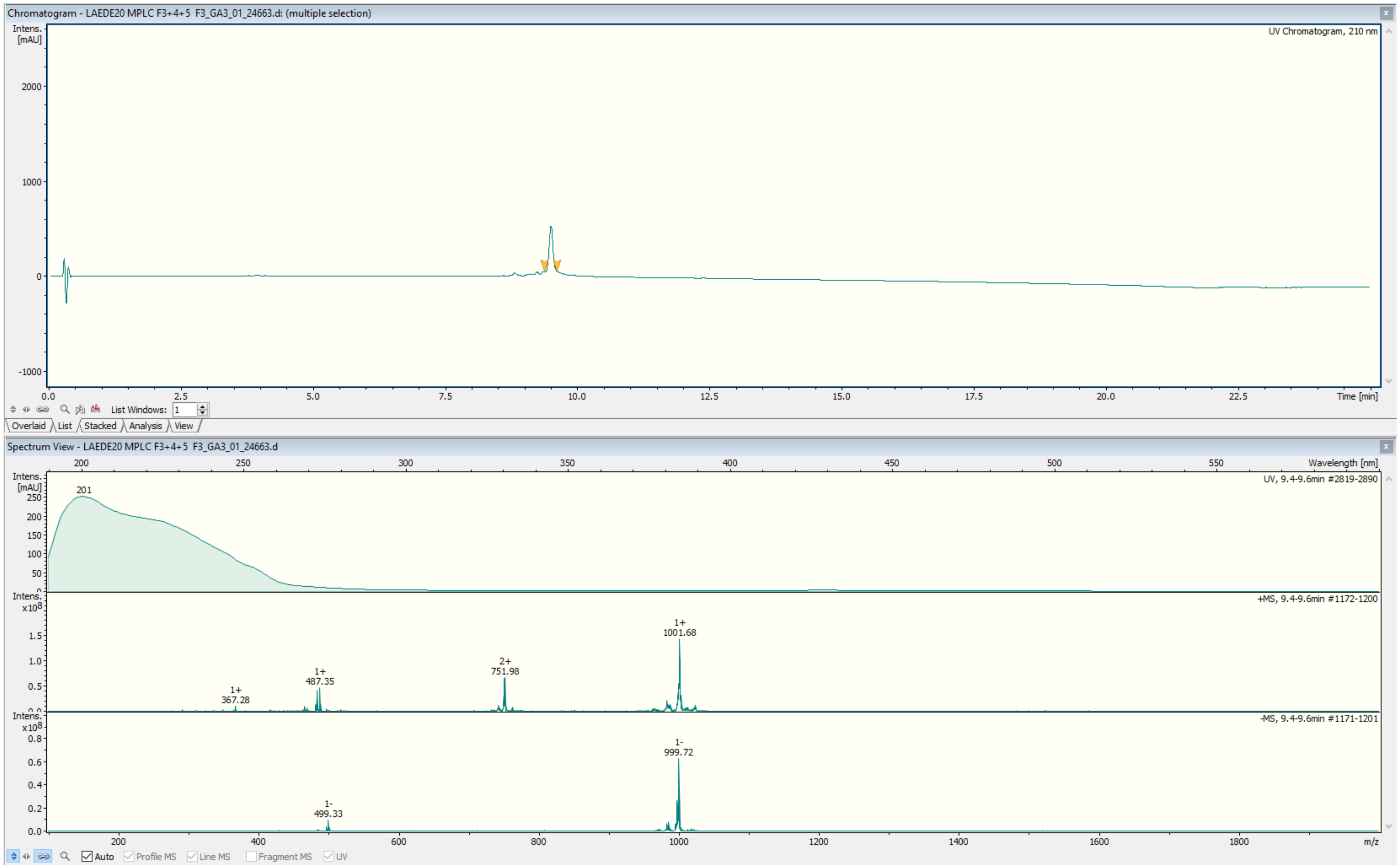

Figure S10: ESIMS data for laetiporin D (**2**)

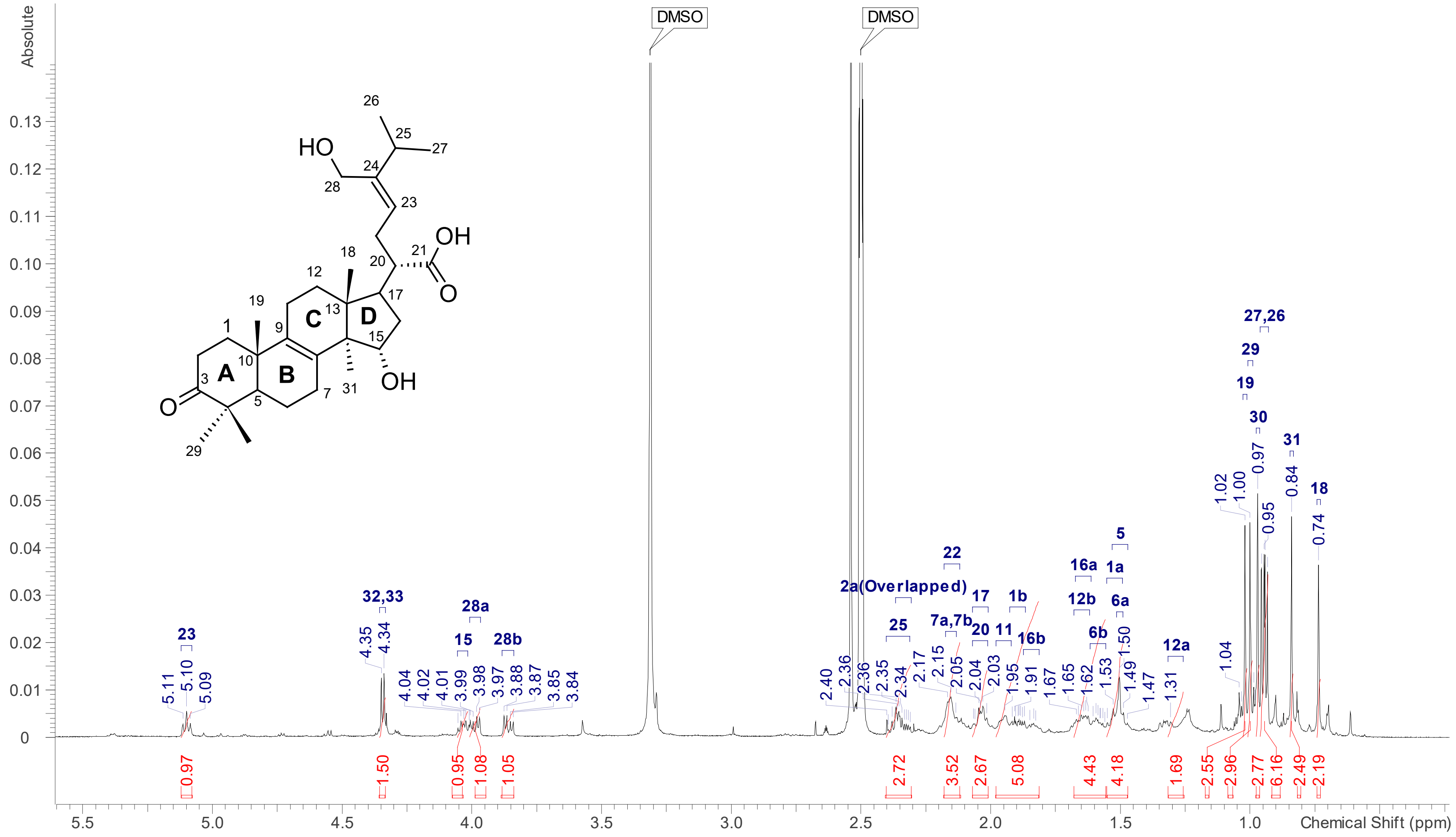

Figure S11: ^{1}H NMR spectrum (DMSO-d_6, 700 MHz) of laetiporin D (**2**).

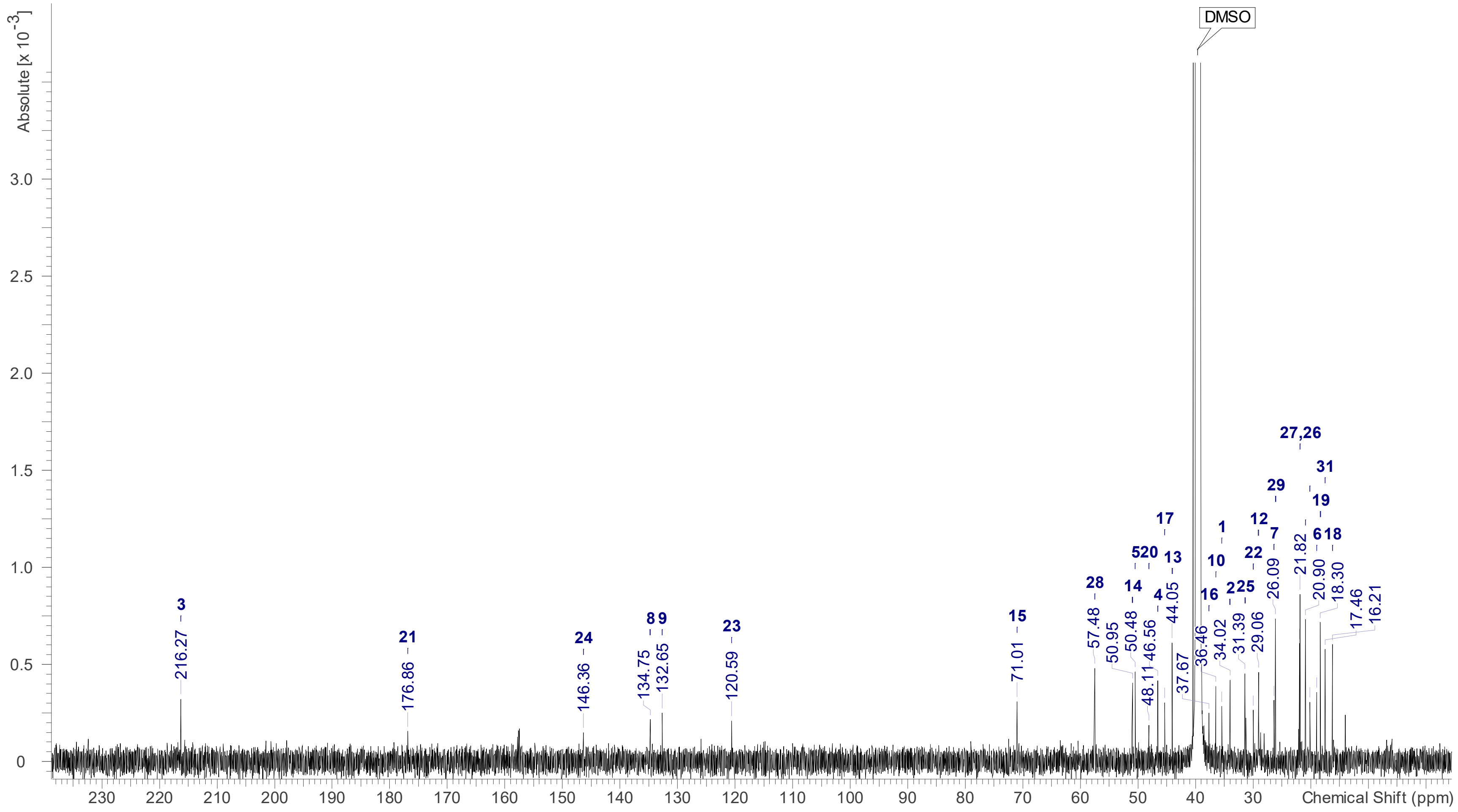

Figure S12: ^{13}C NMR spectrum (DMSO-d_6, 700 MHz) of laetiporin D (**2**).

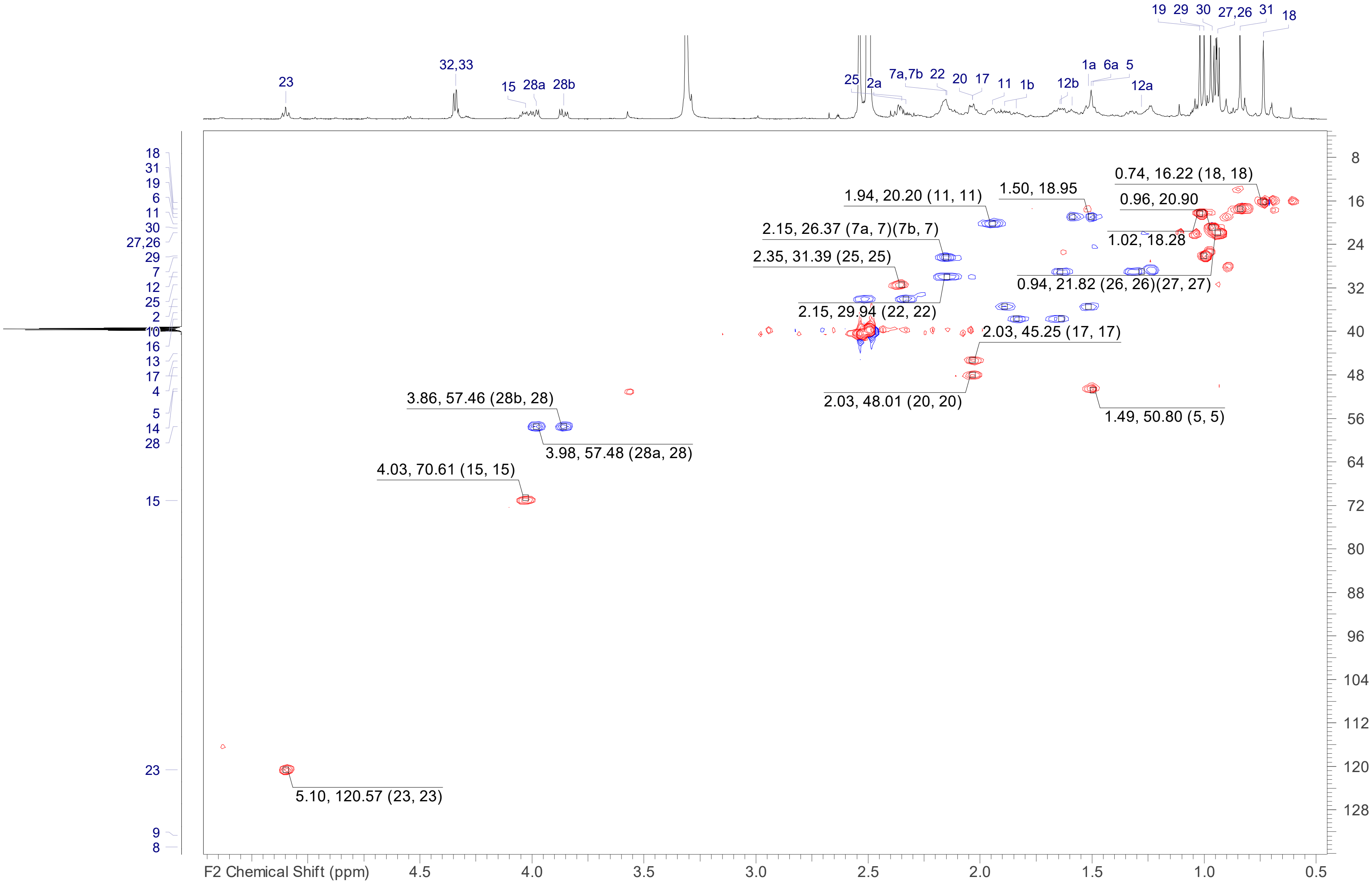

Figure S13: HSQC spectrum (DMSO-d_6, 700 MHz) of laetiporin D (**2**).

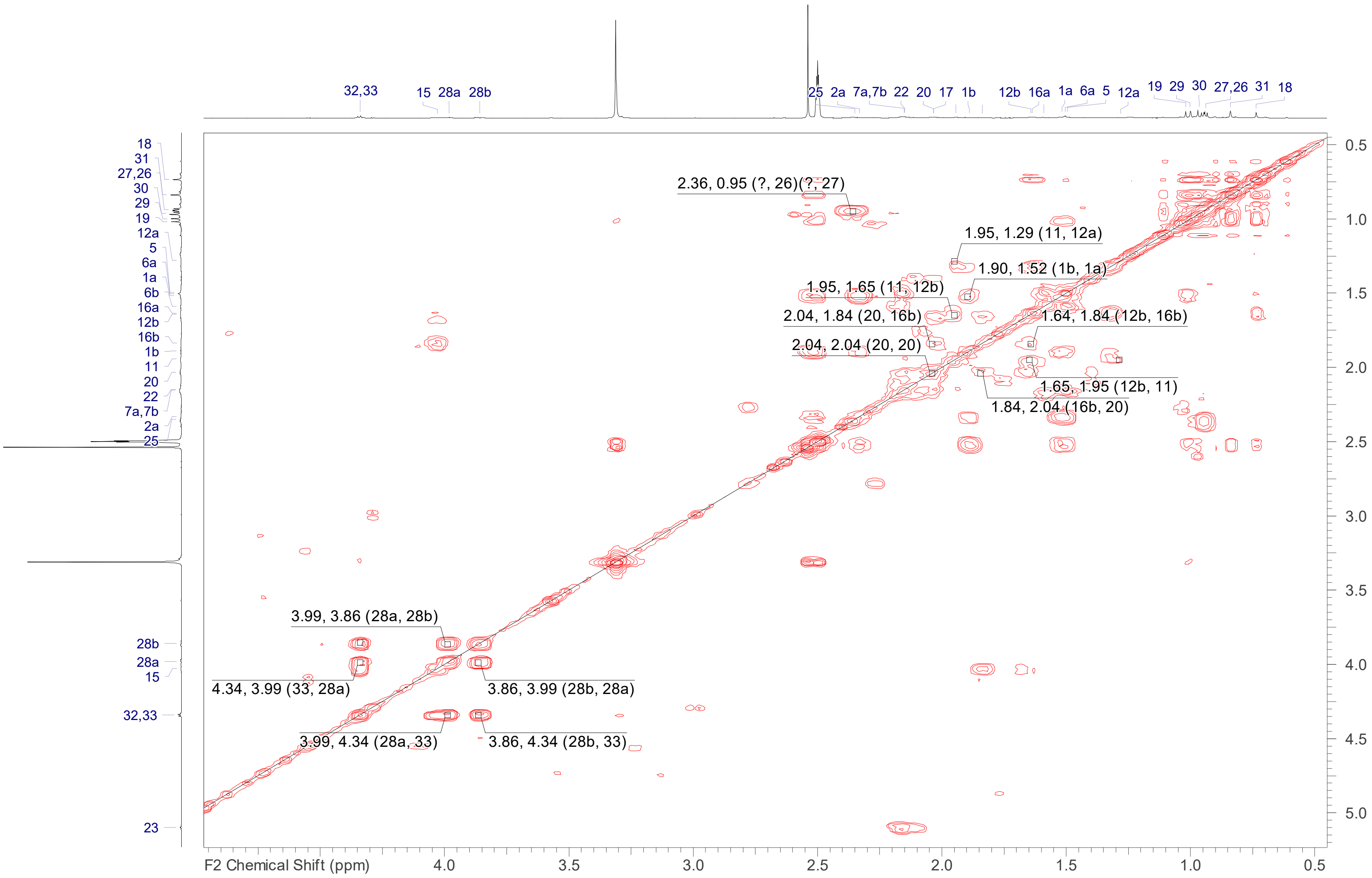

Figure S14: COSY spectrum (DMSO-d_6, 700 MHz) of laetiporin D (**2**).

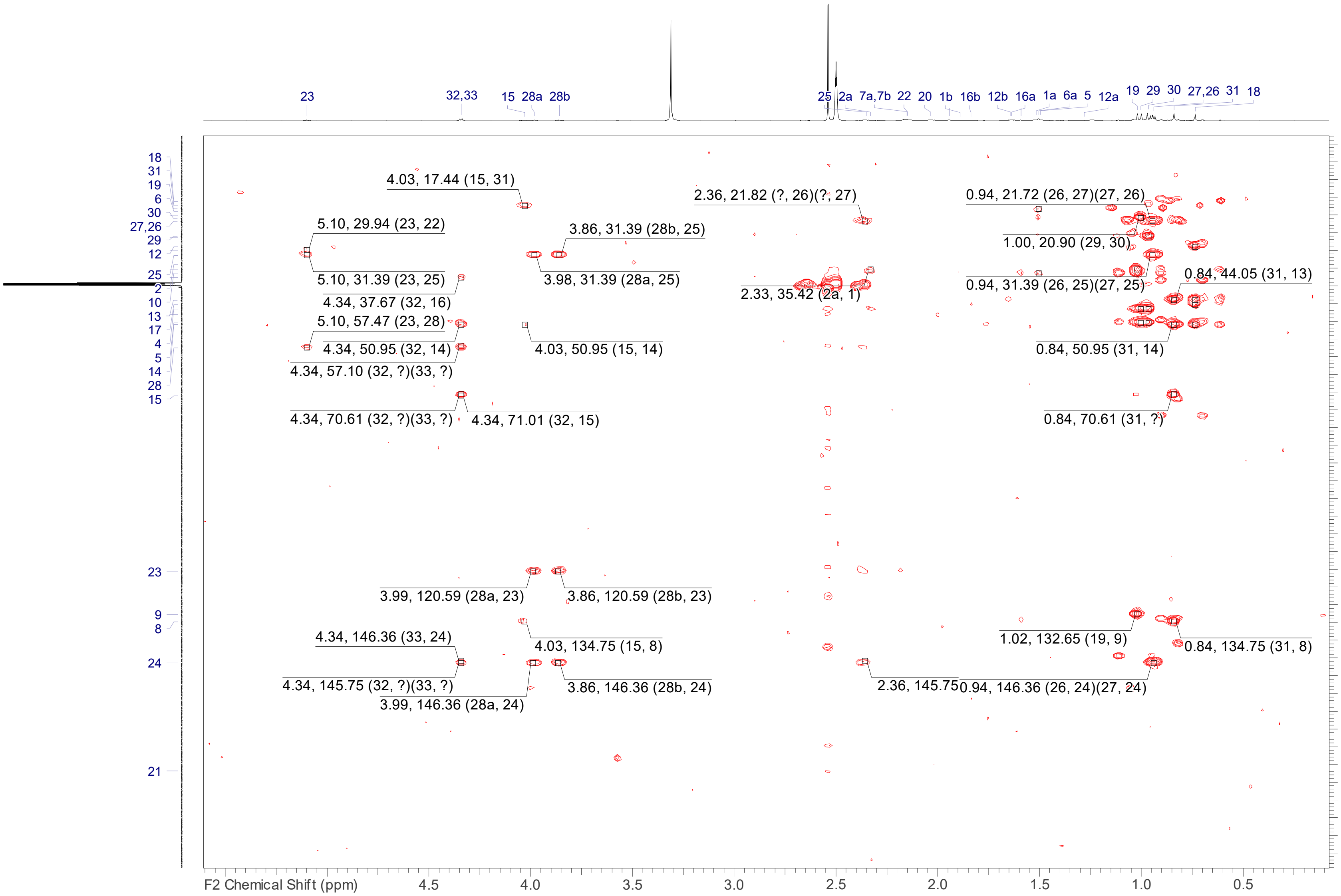

Figure S15: HMBC spectrum (DMSO-d_6, 700 MHz) of laetiporin D (**2**).

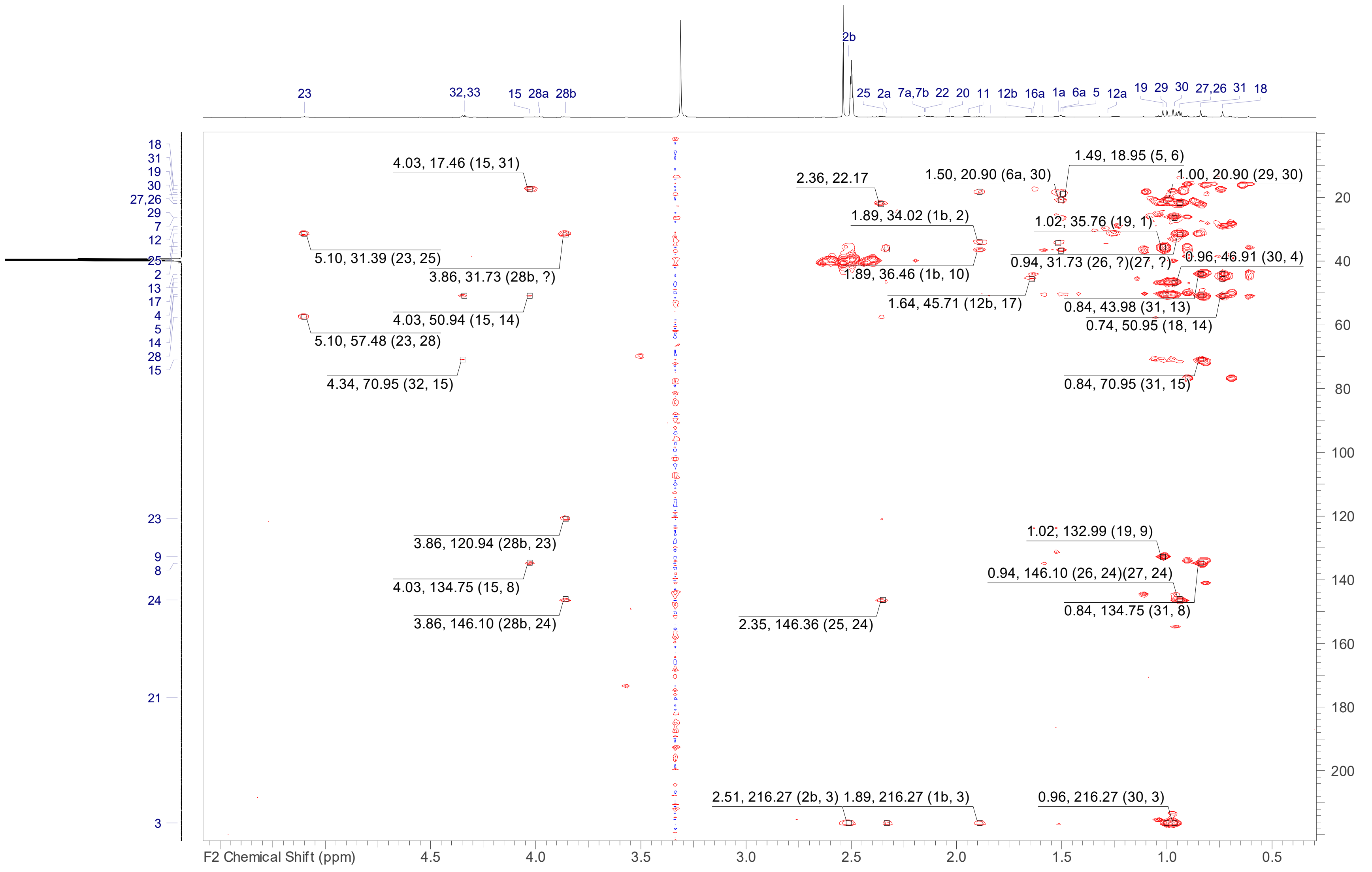

Figure S16: HMBC spectrum (DMSO-*d*$_6$, 700 MHz) of laetiporin D (**2**).

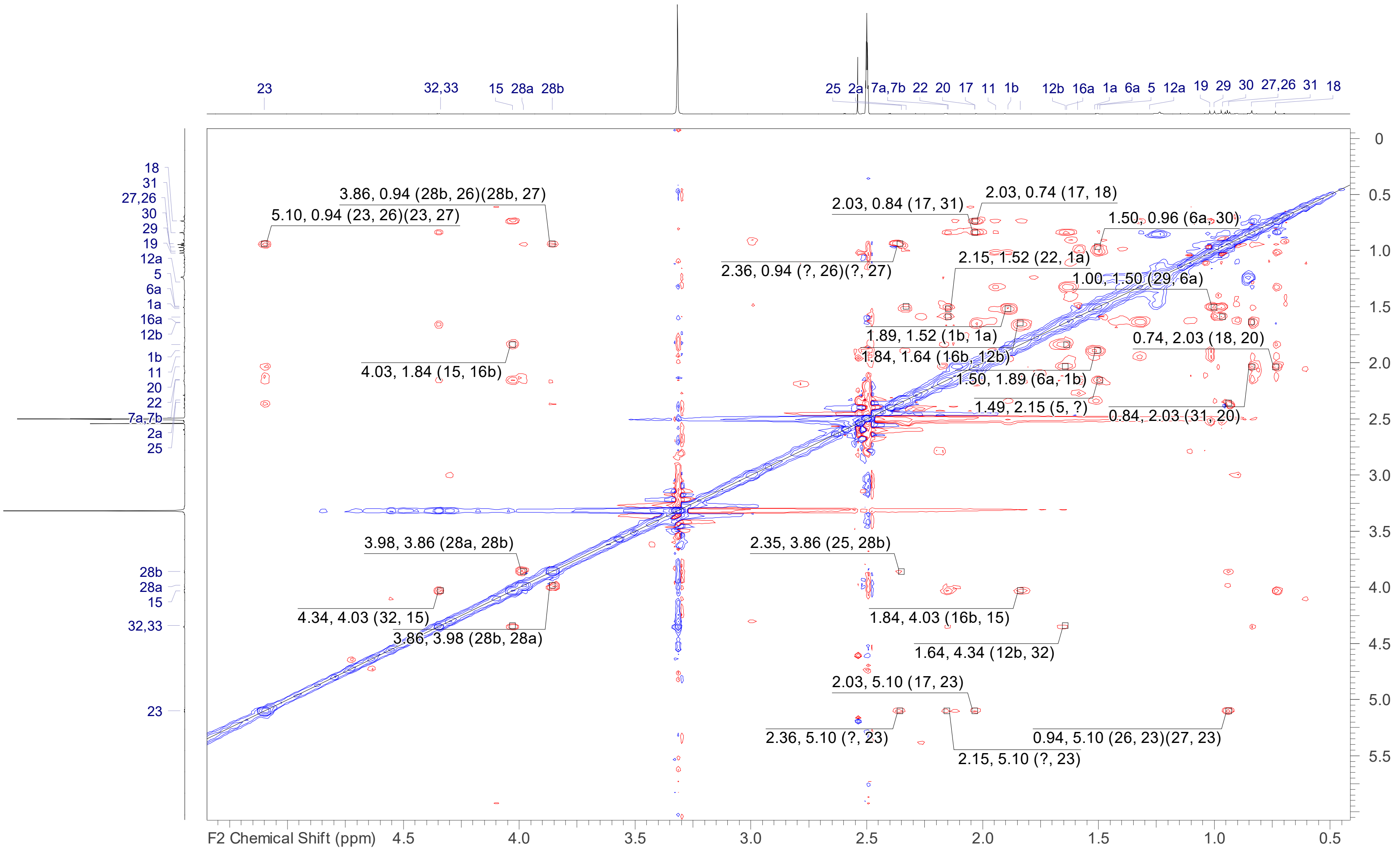

Figure S17: ROESY spectrum (DMSO-d_6, 700 MHz) of laetiporin D (**2**).

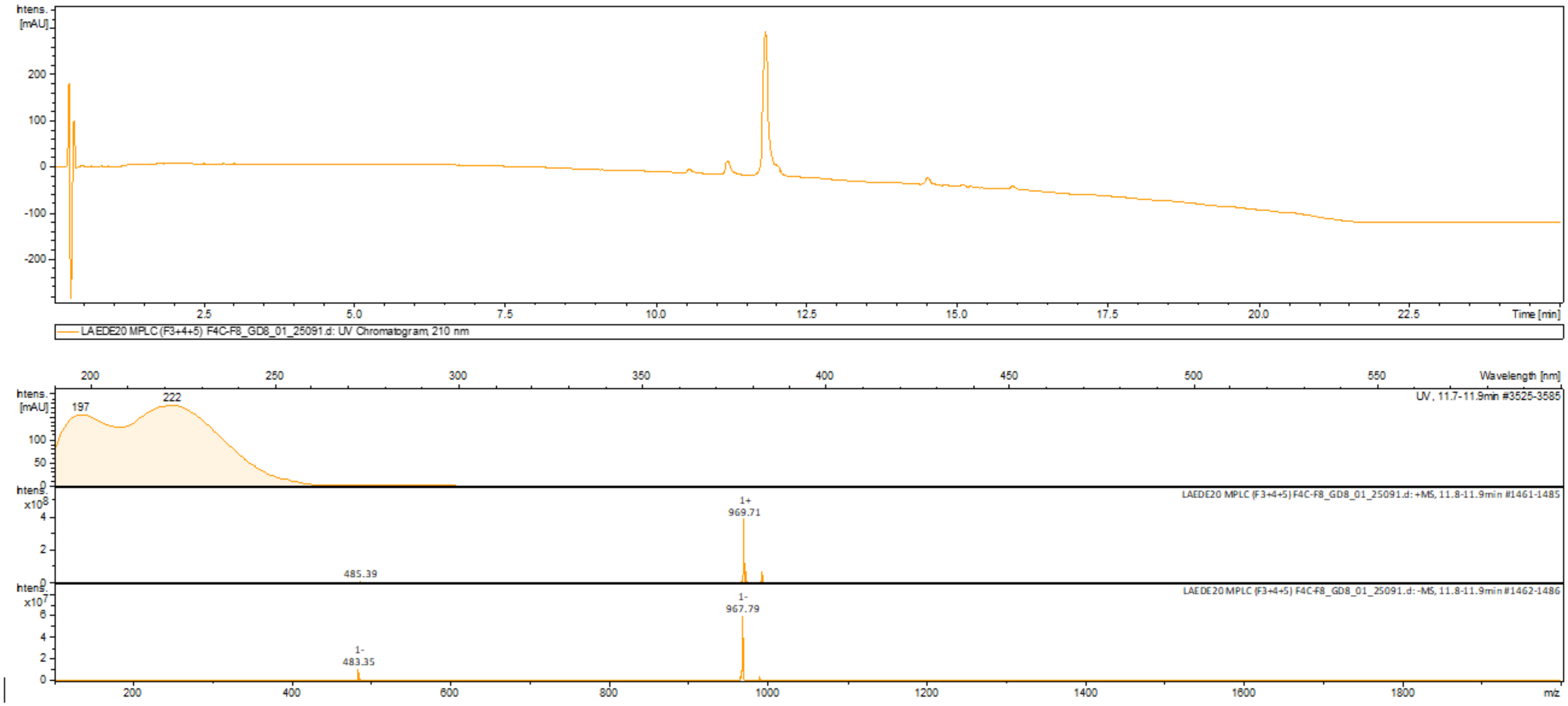

Figure S18: ESIMS data for fomefficinic acid D (**3**)

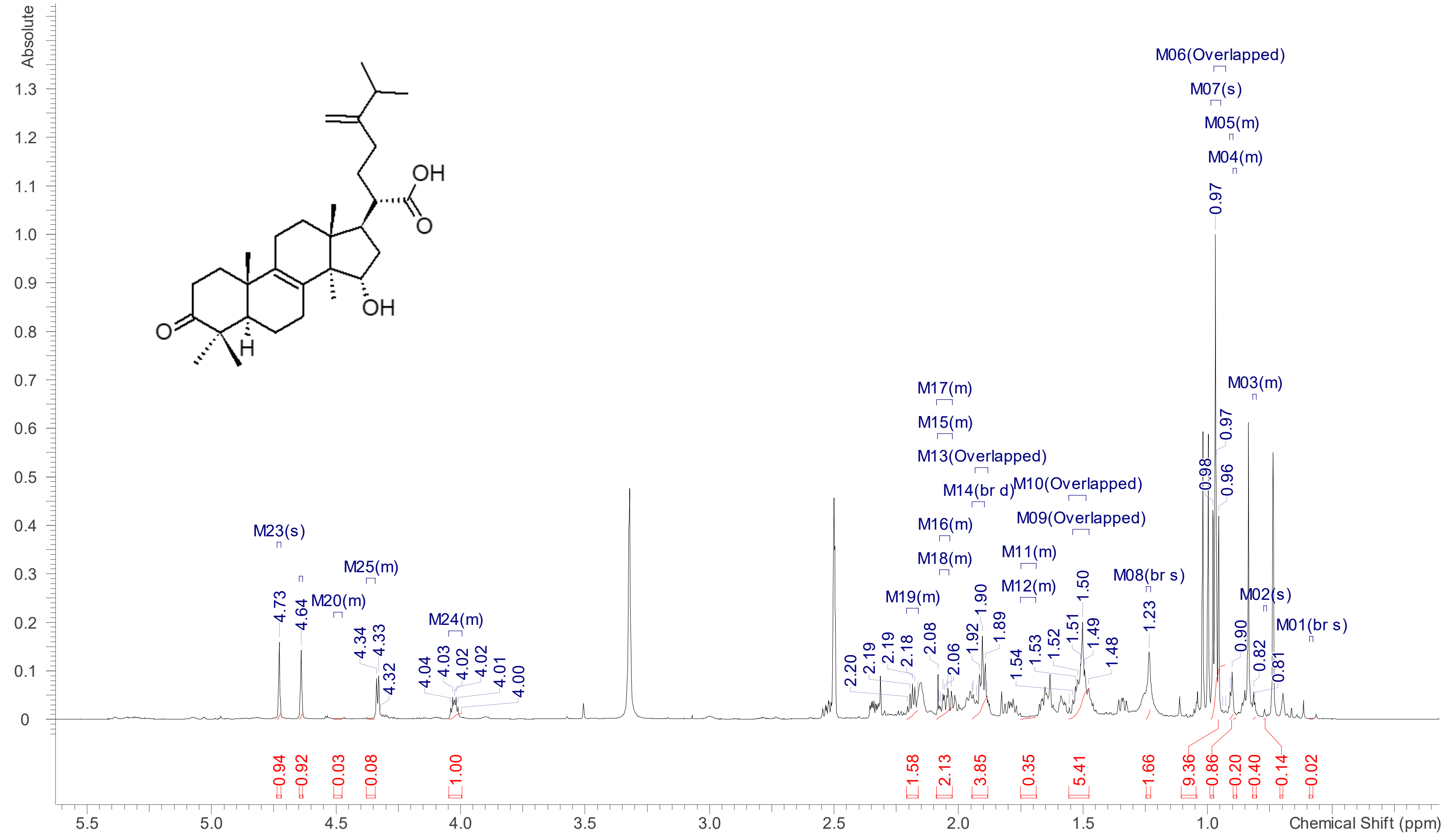

Figure S19: ^{1}H NMR spectrum (DMSO-d_6, 700 MHz) of fomefficinic acid D (**3**).

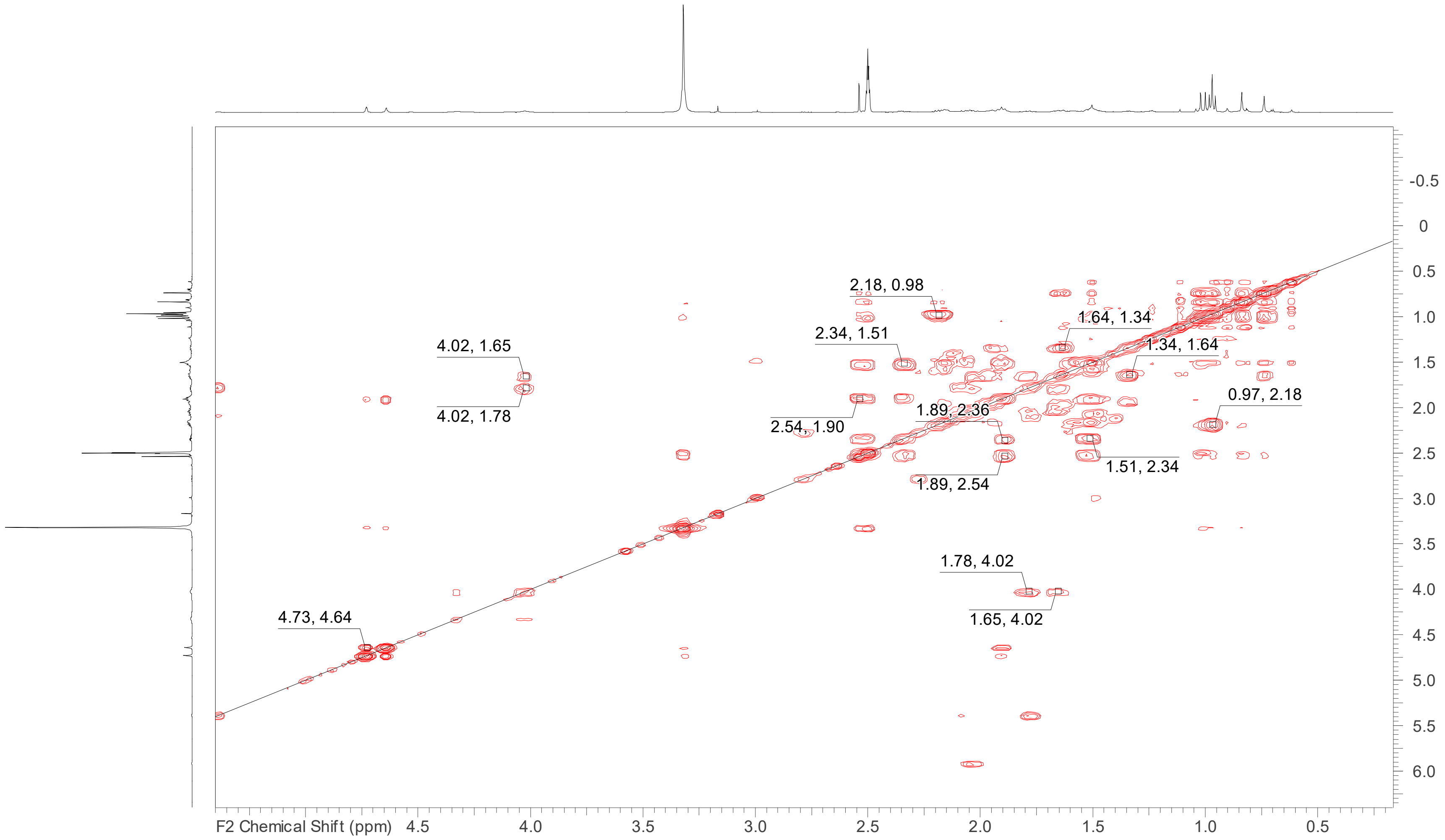

Figure S20: COSY spectrum (DMSO-d_6, 700 MHz) of fomefficinic acid D (**3**).

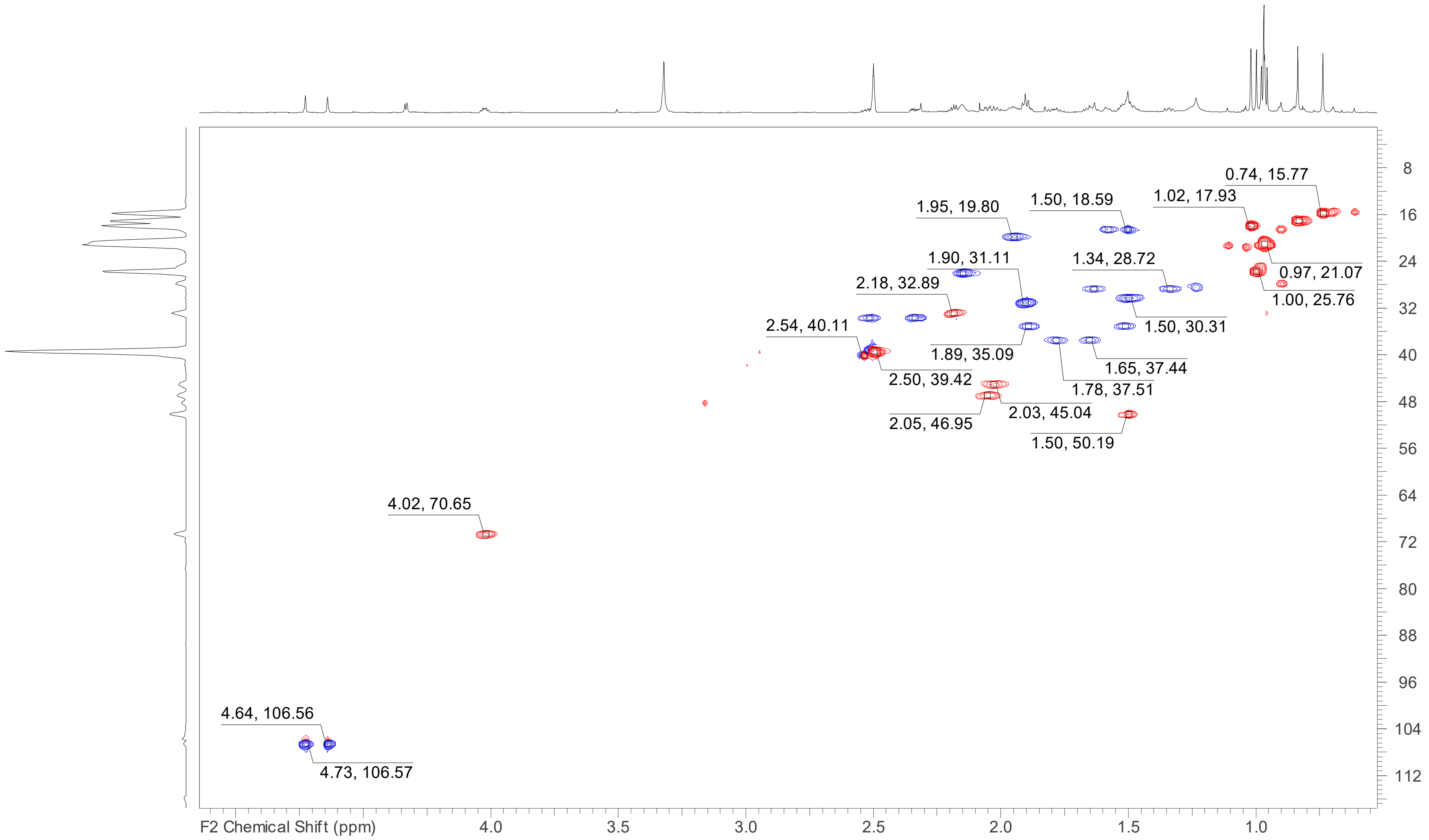

Figure S21: HSQC spectrum (DMSO-d_6, 700 MHz) of fomefficinic acid D (**3**).

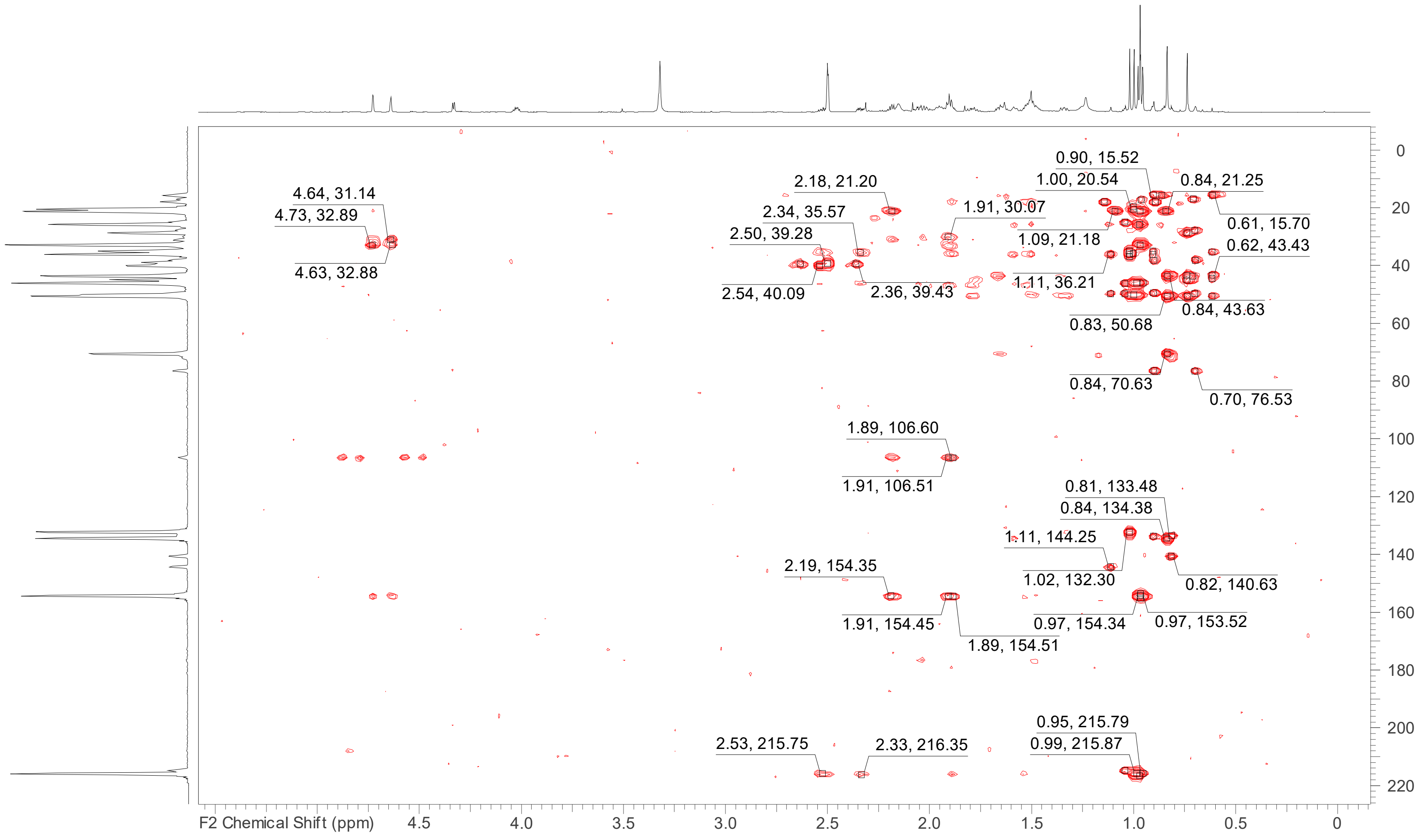

Figure S22: HMBC spectrum (DMSO-d_6, 700 MHz) of fomefficinic acid D (**3**).

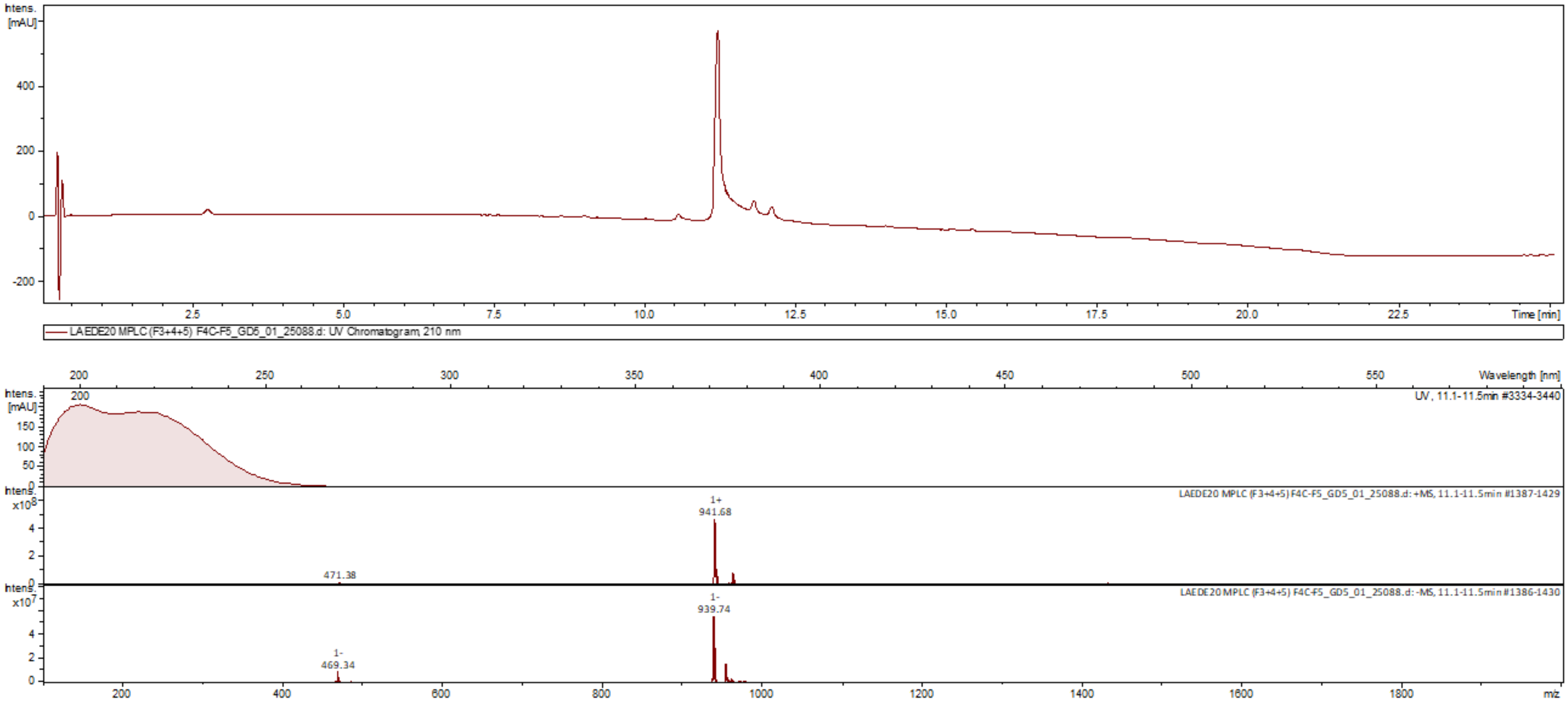

Figure S23:ESIMS data of eburicoic acid (**4**)

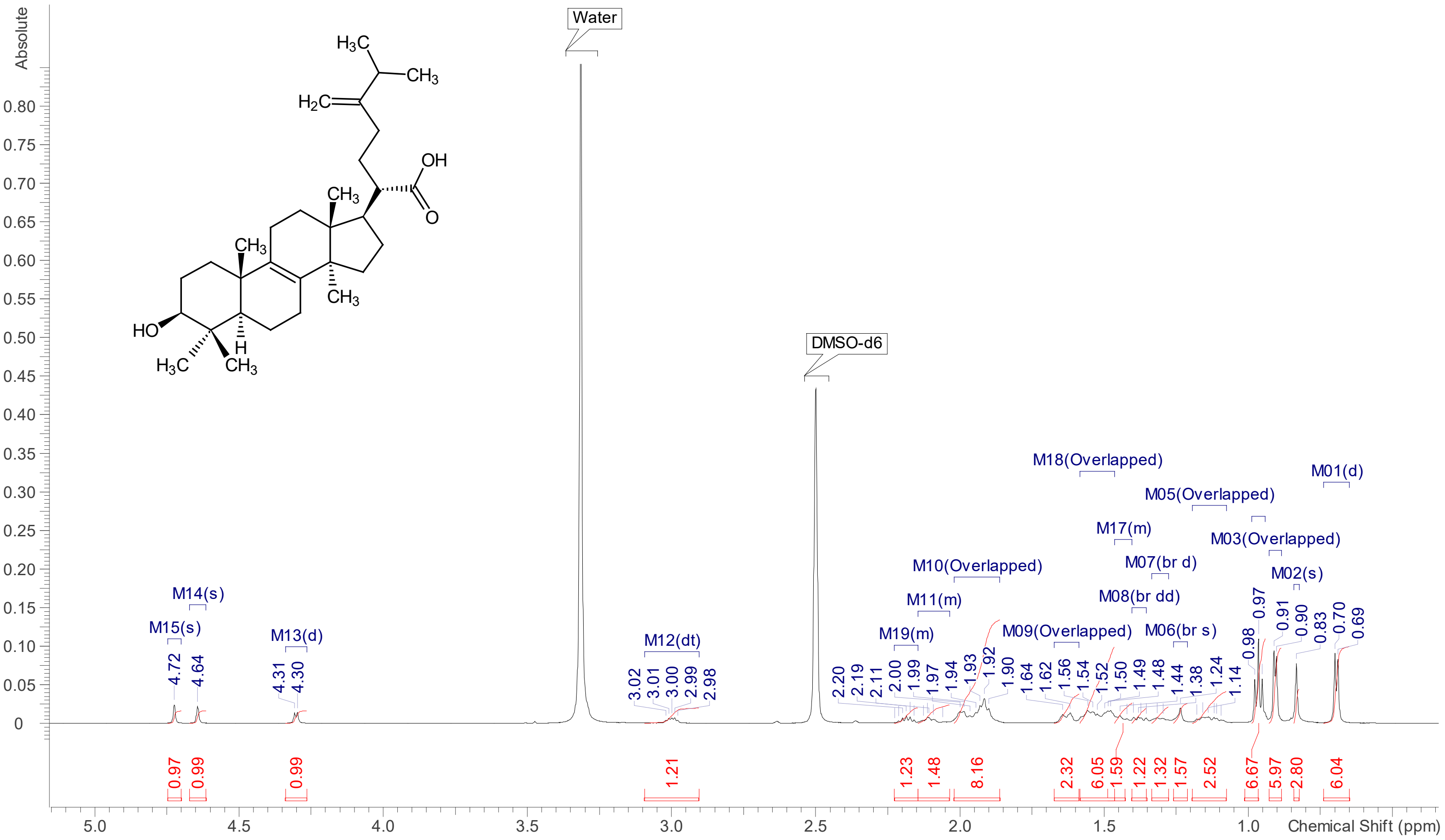

Figure S24: ^{1}H NMR spectrum (DMSO-d_6, 700 MHz) of eburicoic acid (**4**)

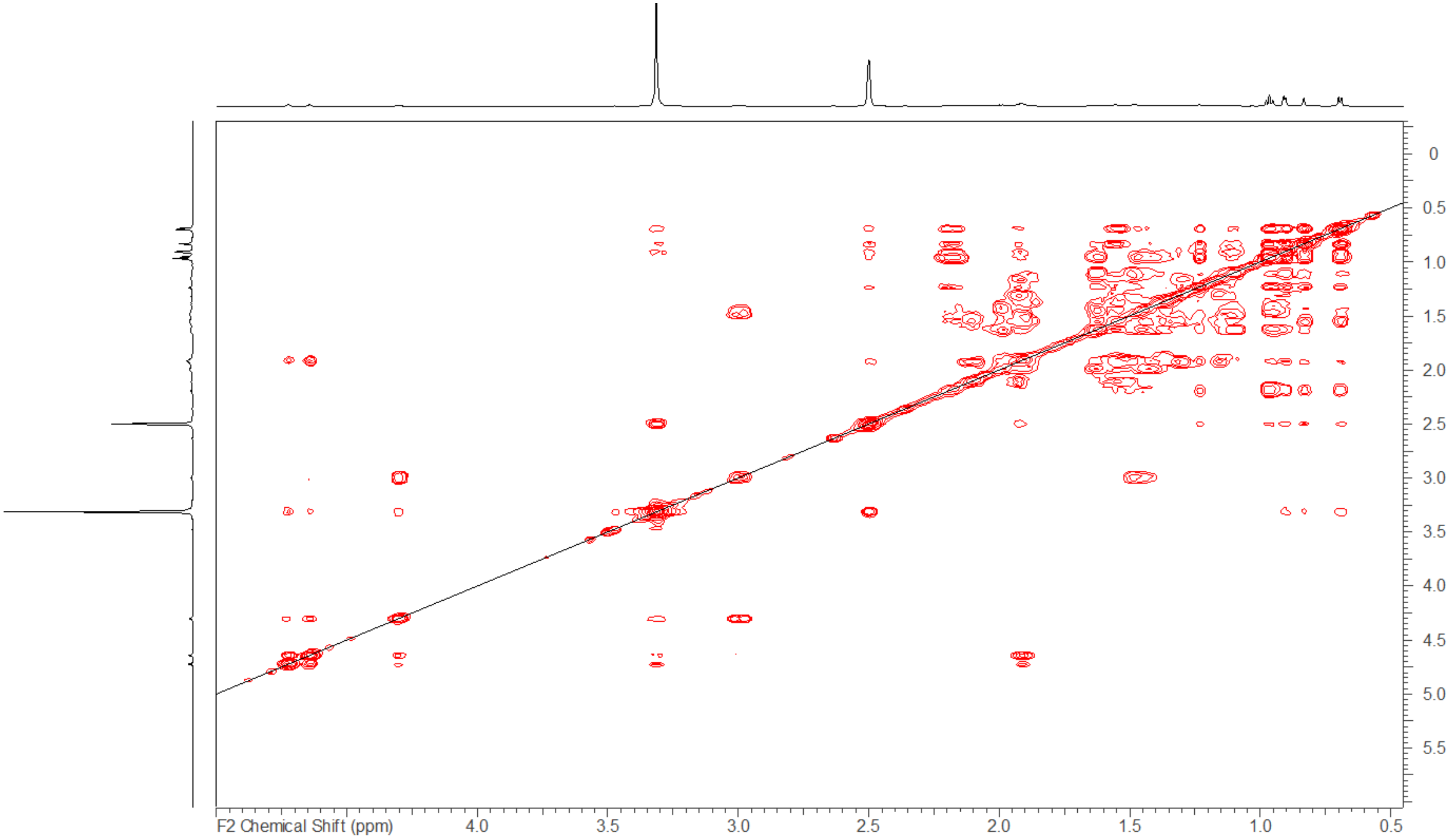

Figure S25: COSY spectrum (DMSO-d_6, 700 MHz) of eburicoic acid (**4**)

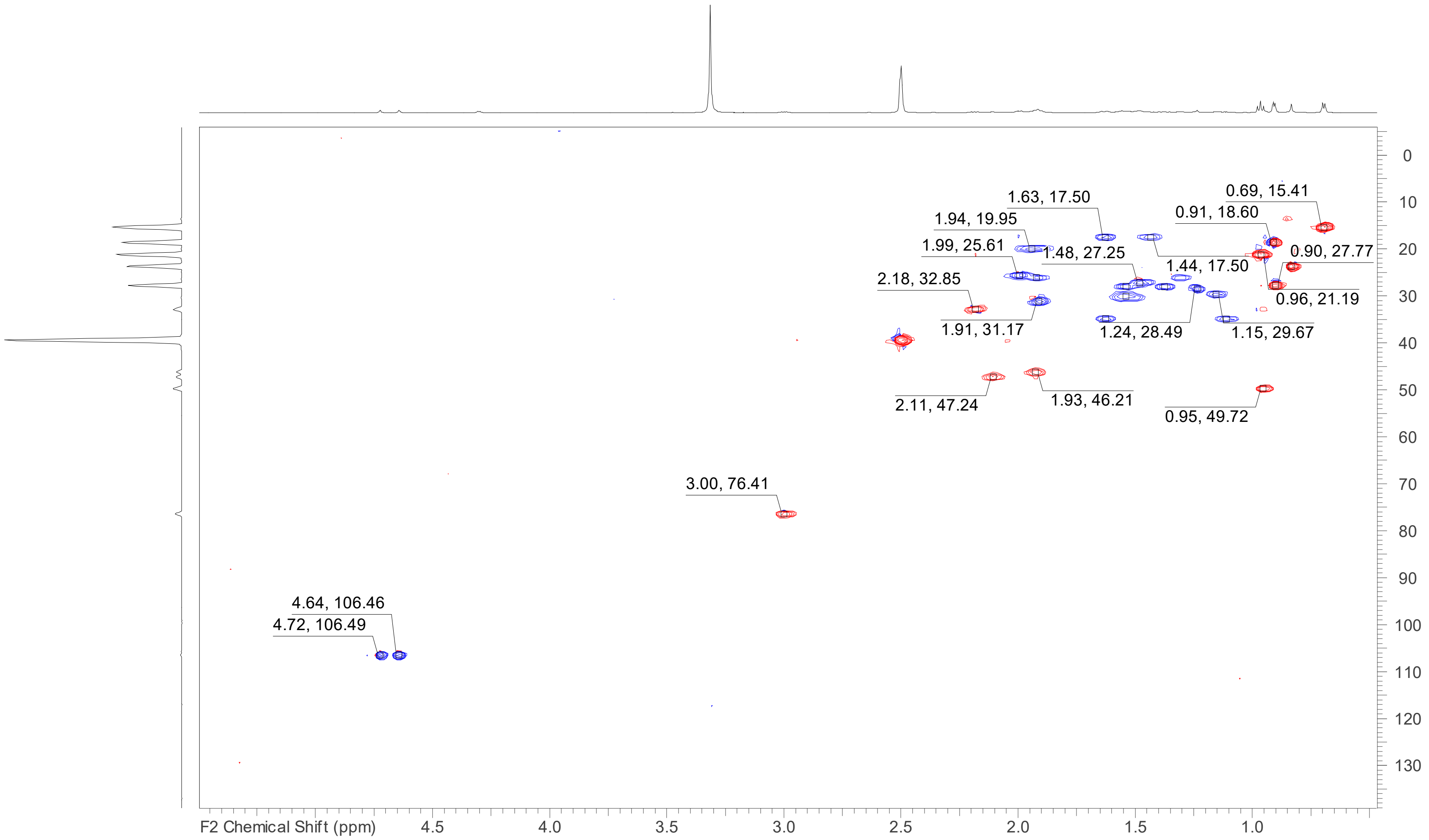

Figure S26: HSQC spectrum (DMSO-d_6, 700 MHz) of eburicoic acid (**4**)

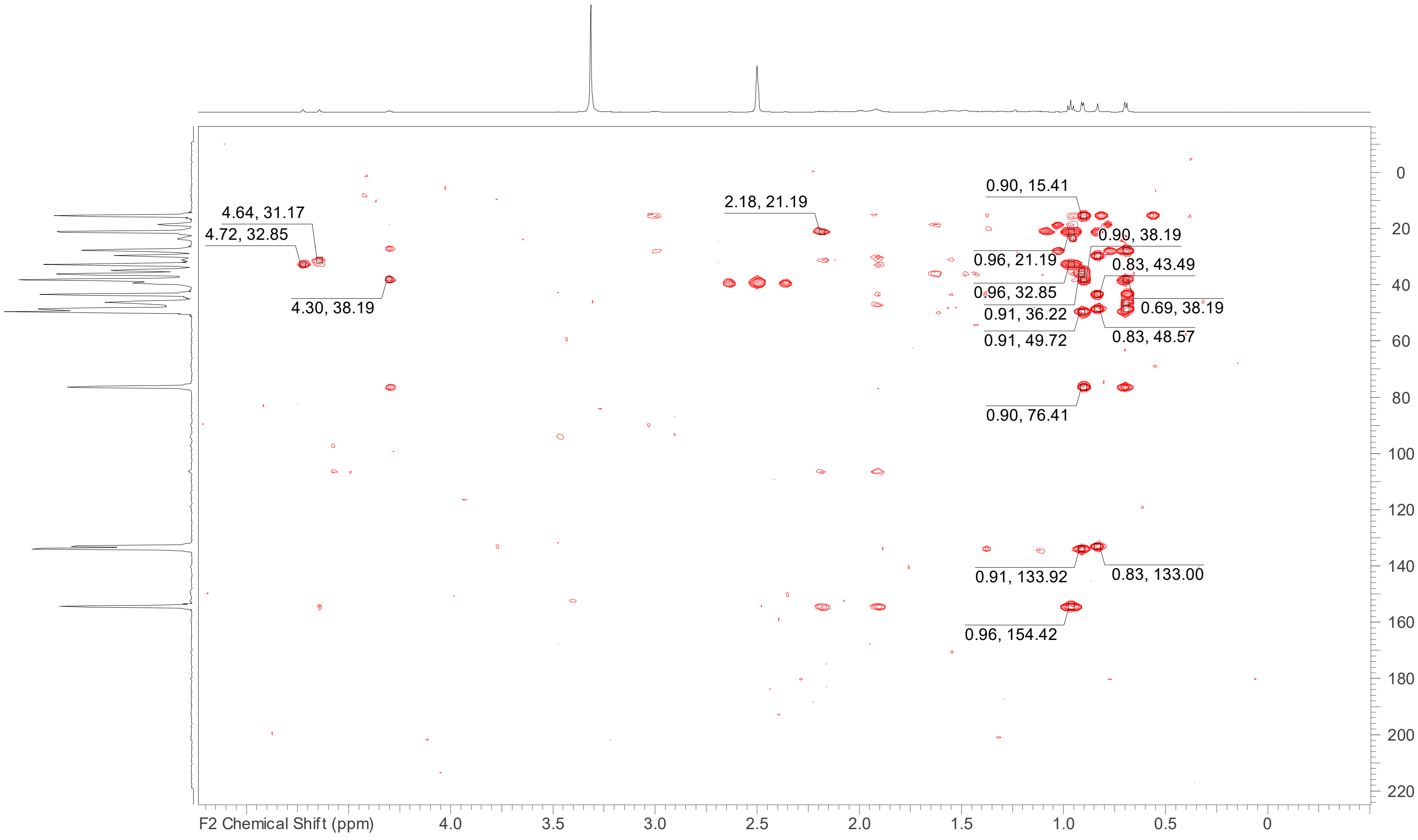

Figure S27: HMBC spectrum (DMSO-d_6, 700 MHz) of eburicoic acid (**4**)

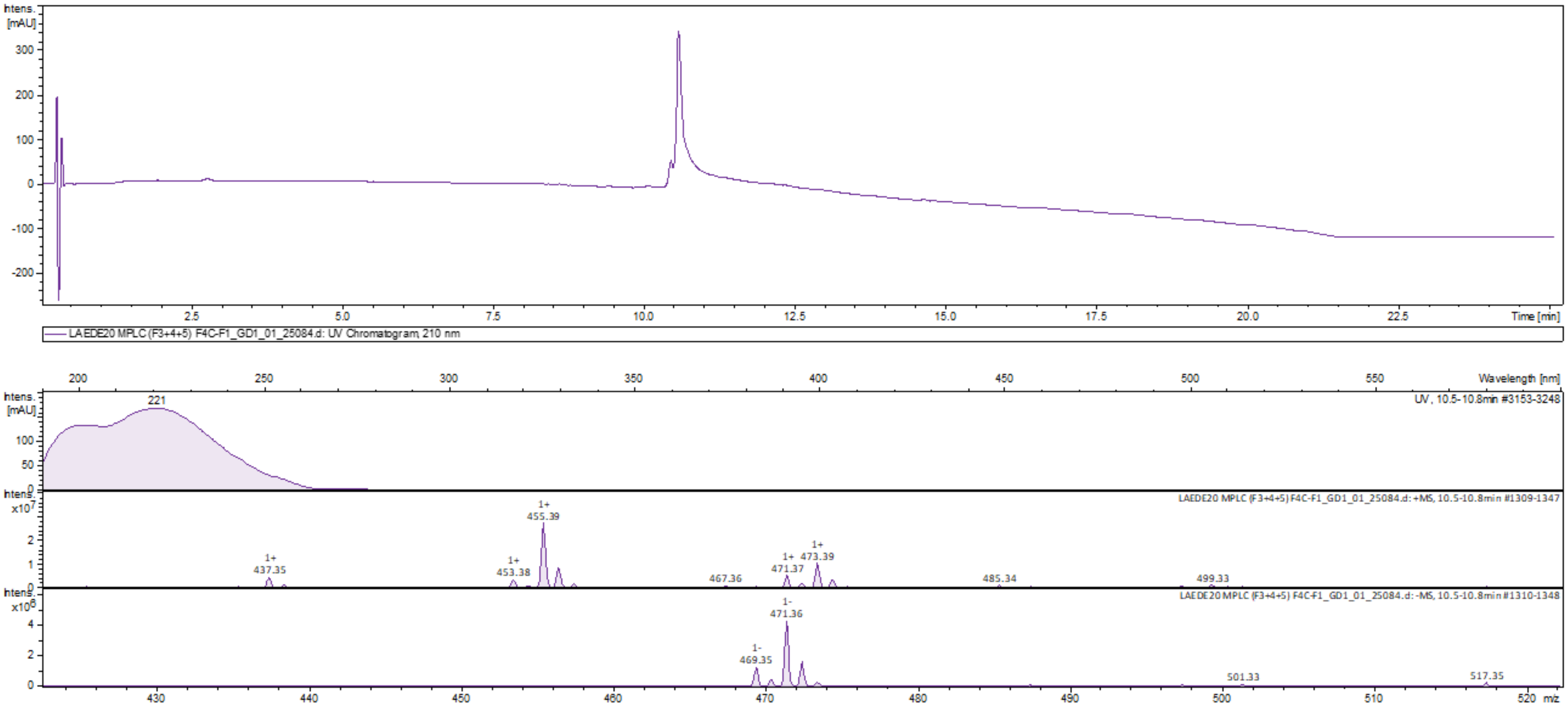

Figure S28: ESIMS data of 15α-hydroxytrametenolic acid (**5**)

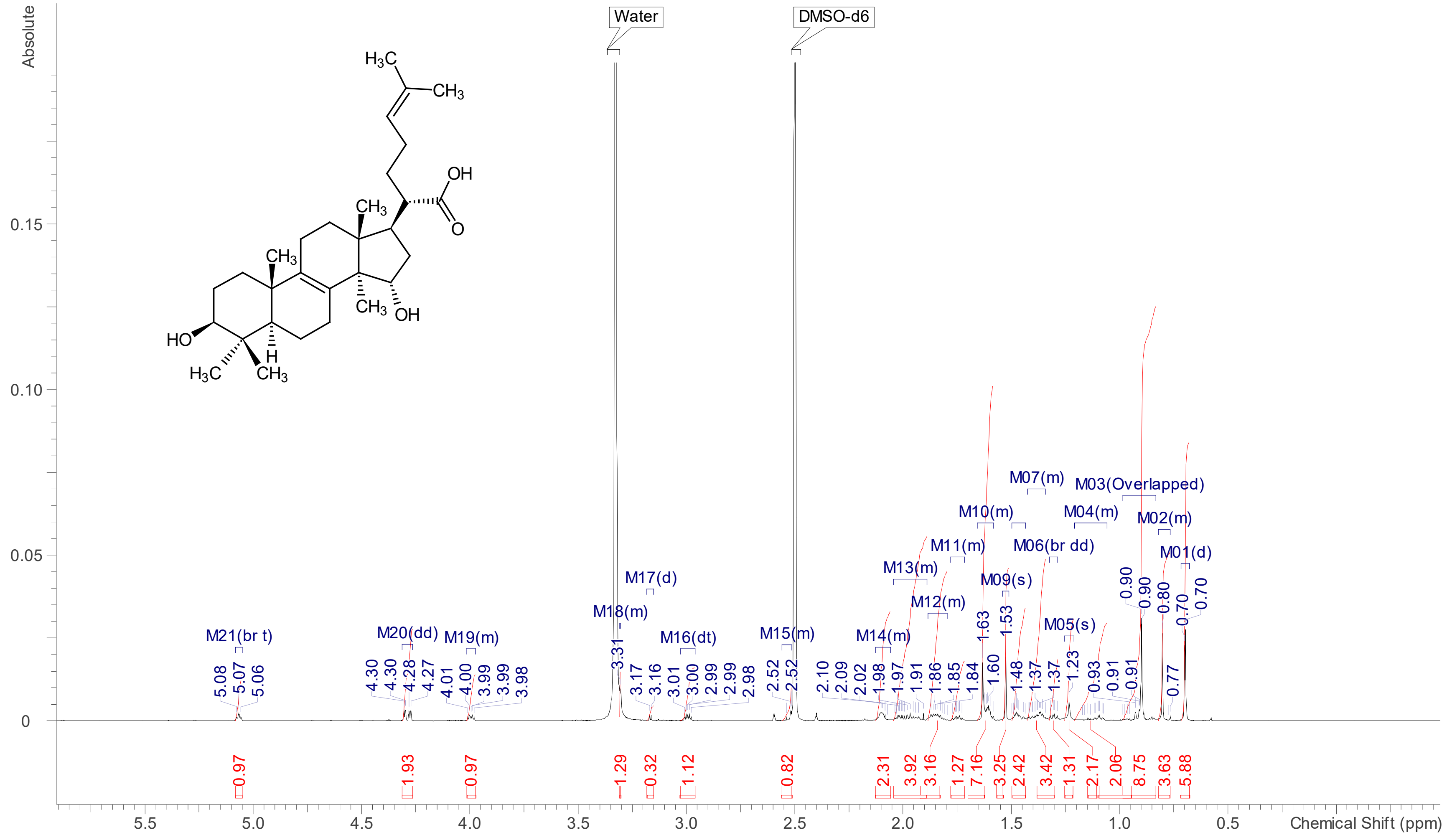

Figure S29: ^{1}H NMR spectrum (DMSO-d_6, 700 MHz) of 15α-hydroxytrametenolic acid (**5**)

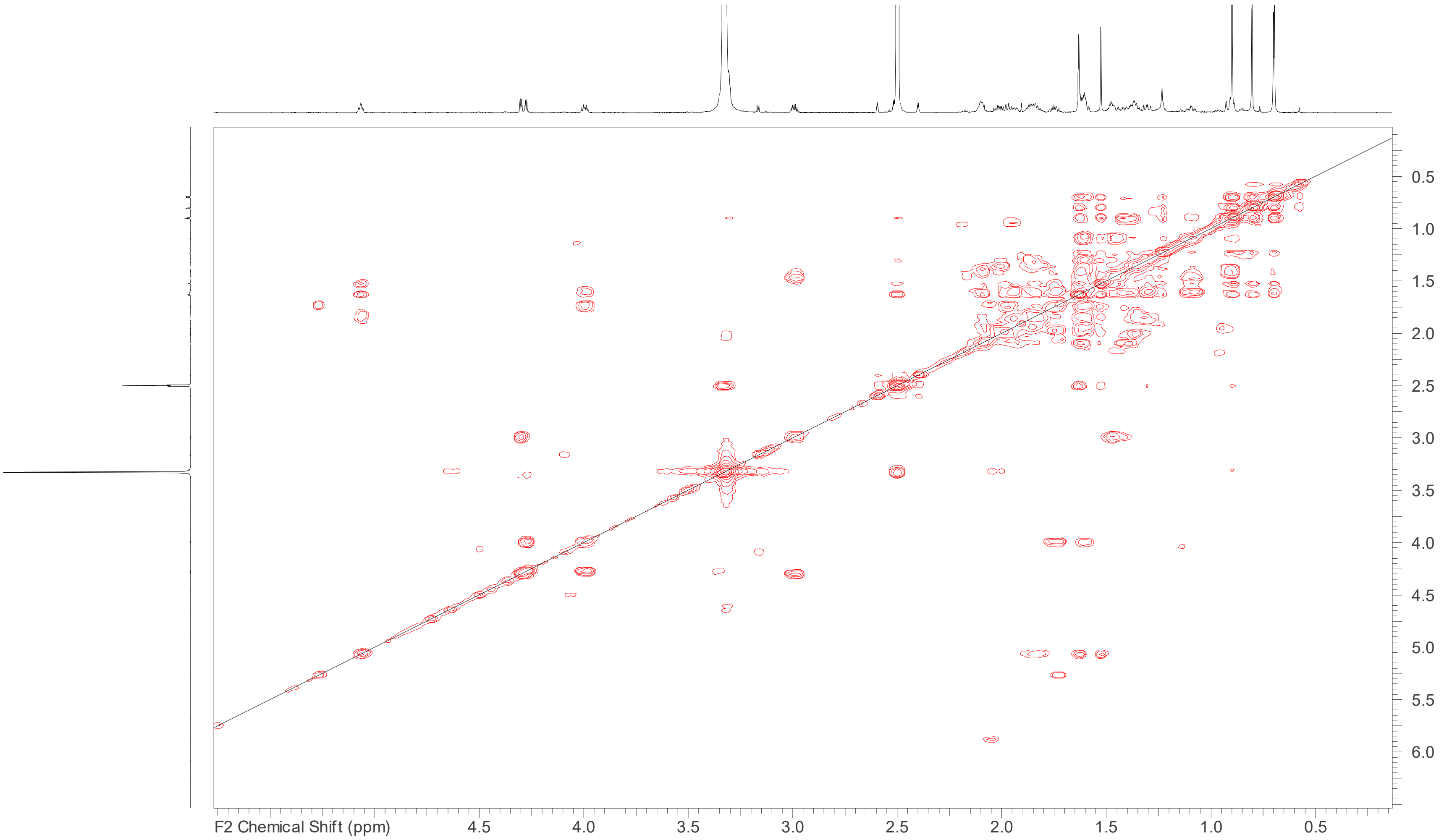

Figure S30: COSY spectrum (DMSO-d_6, 700 MHz) of 15α-hydroxytrametenolic acid (**5**)

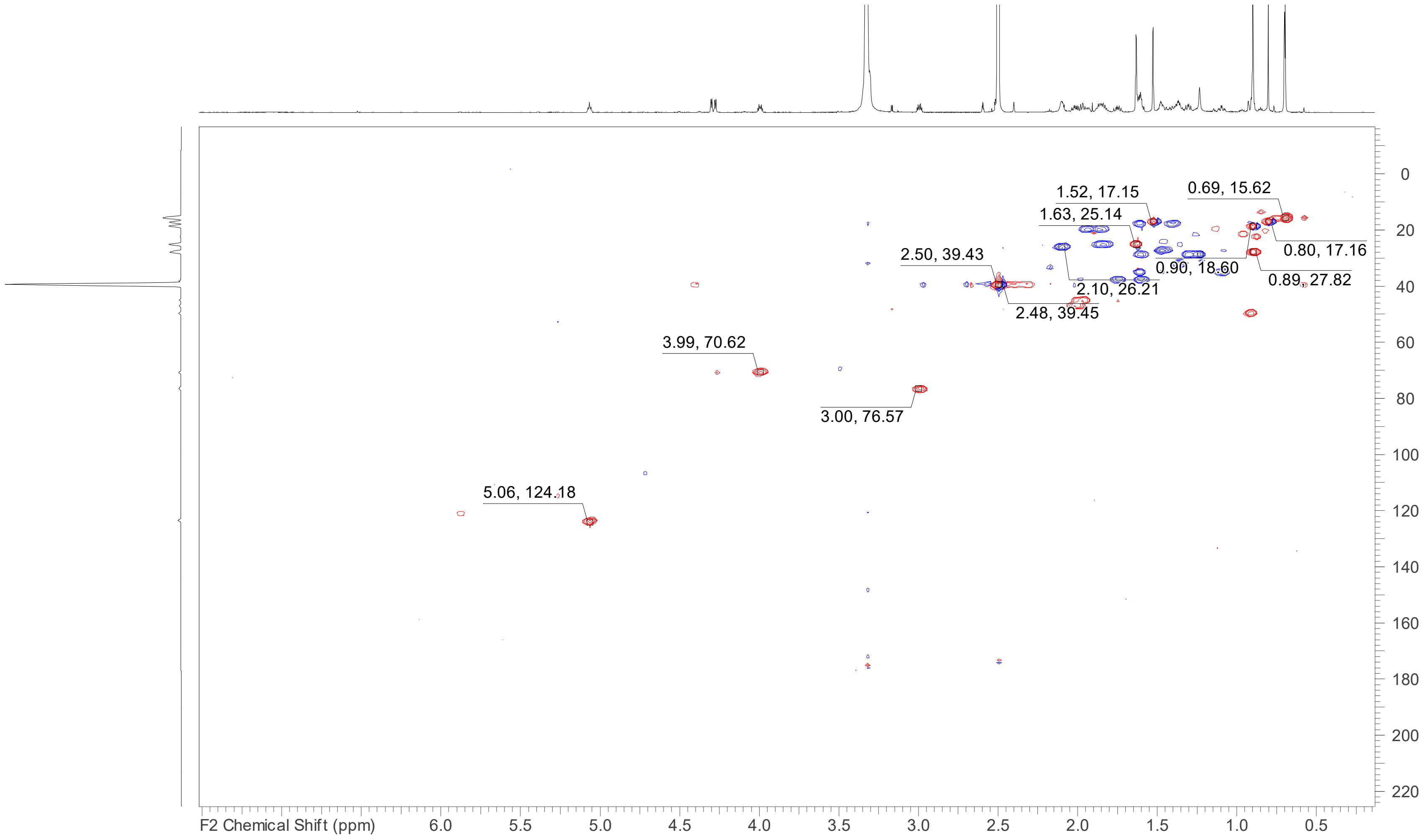

Figure S31: HSQC spectrum (DMSO-d_6, 700 MHz) of 15α-hydroxytrametenolic acid (**5**)

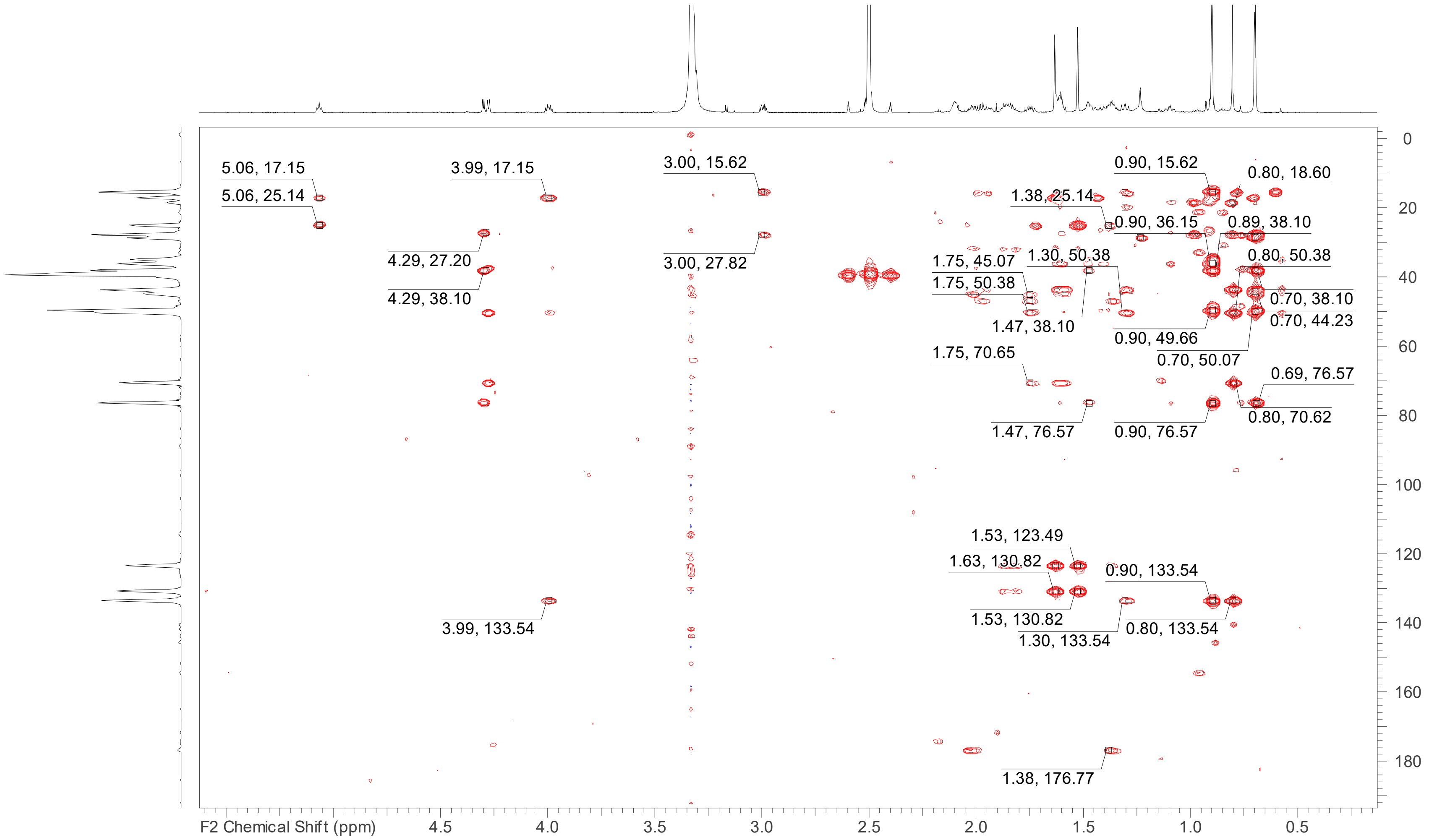

Figure S32: HMBC spectrum (DMSO-d_6, 700 MHz) of 15α-hydroxytrametenolic acid (**5**)

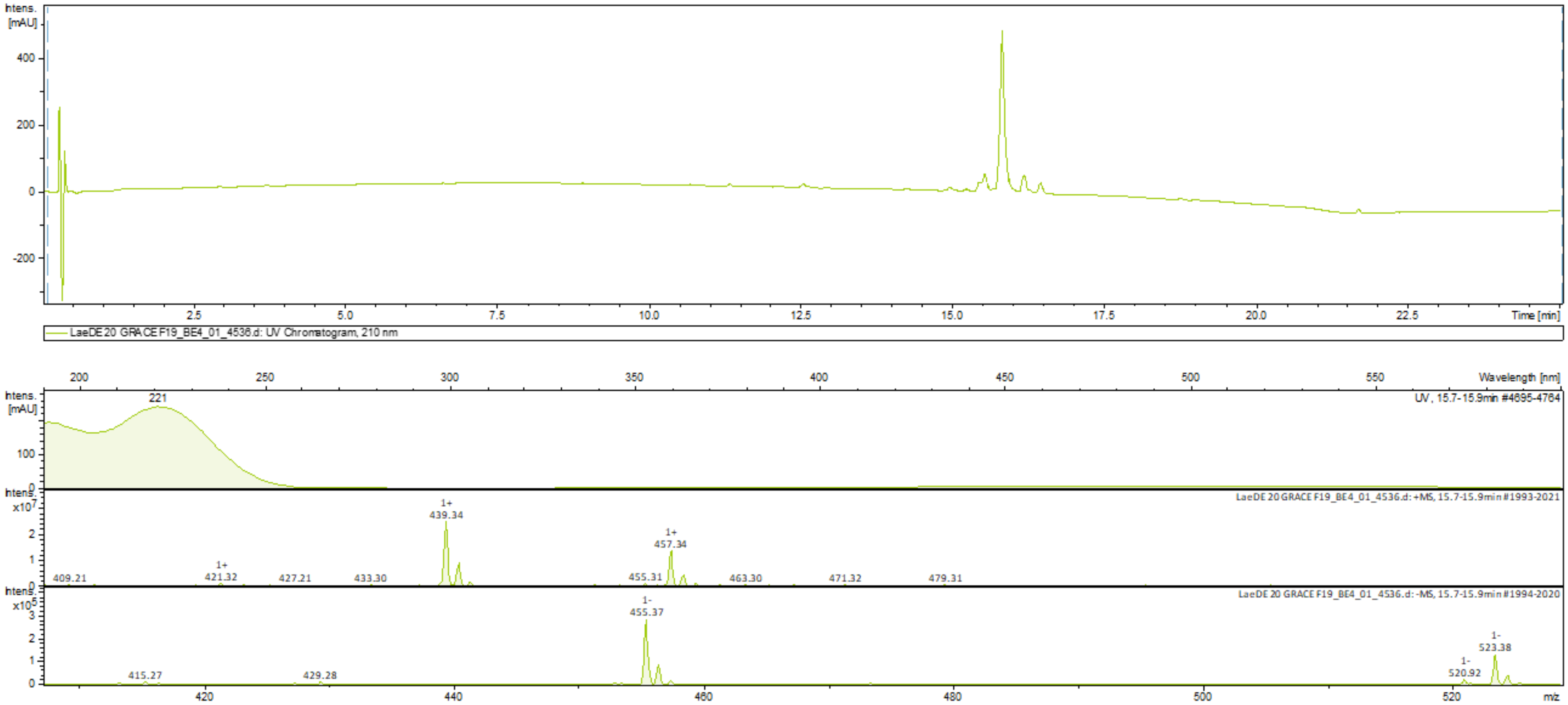

Figure S33: ESIMS data of trametenolic acid (**6**)

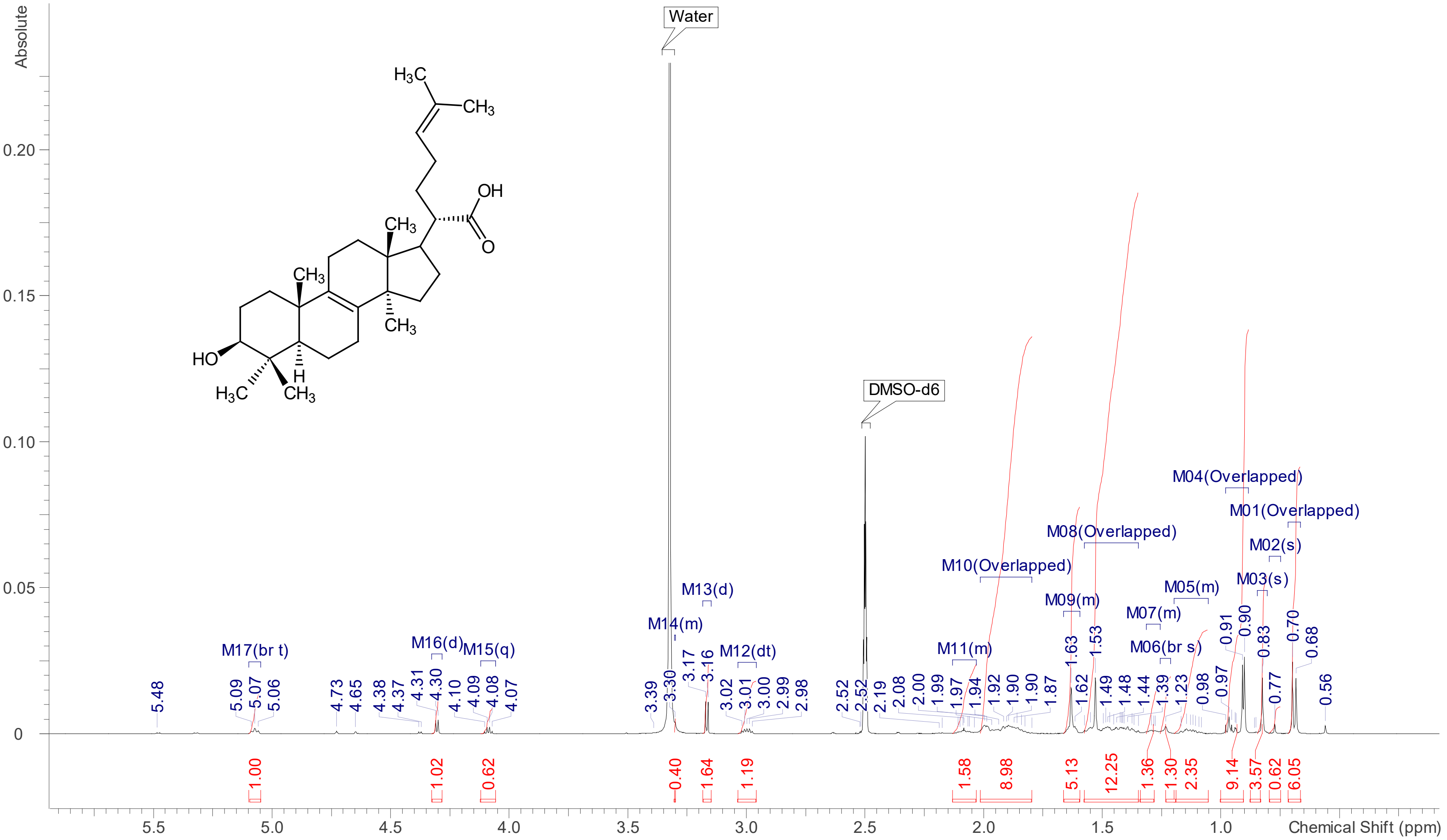

Figure S34: ^{1}H NMR spectrum (DMSO-d_6, 700 MHz) of trametenolic acid (**6**)

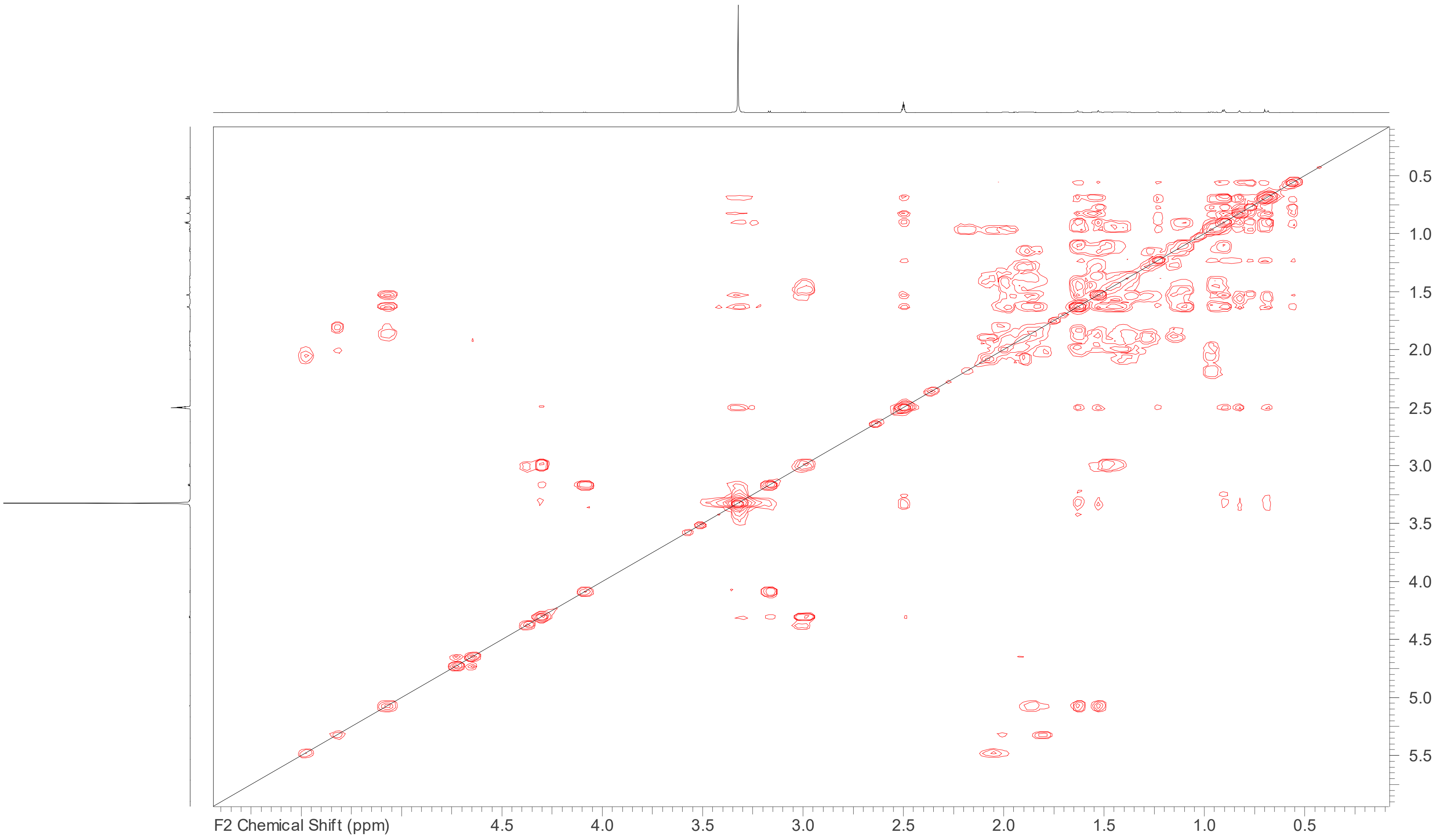

Figure S35: COSY spectrum (DMSO-d_6, 700 MHz) of trametenolic acid (**6**)

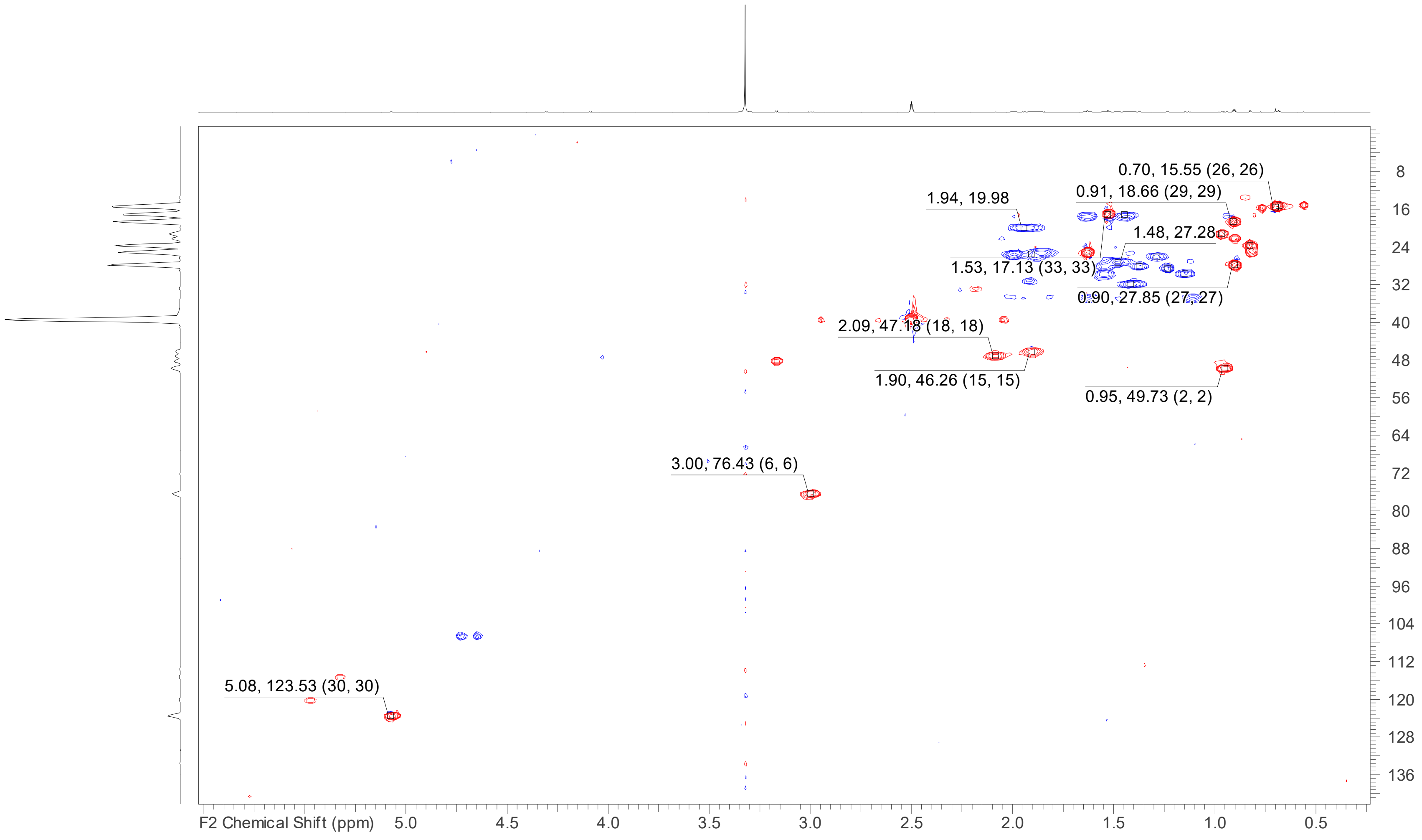

Figure S36: HSQC spectrum (DMSO-*d*6, 700 MHz) of trametenolic acid (**6**)

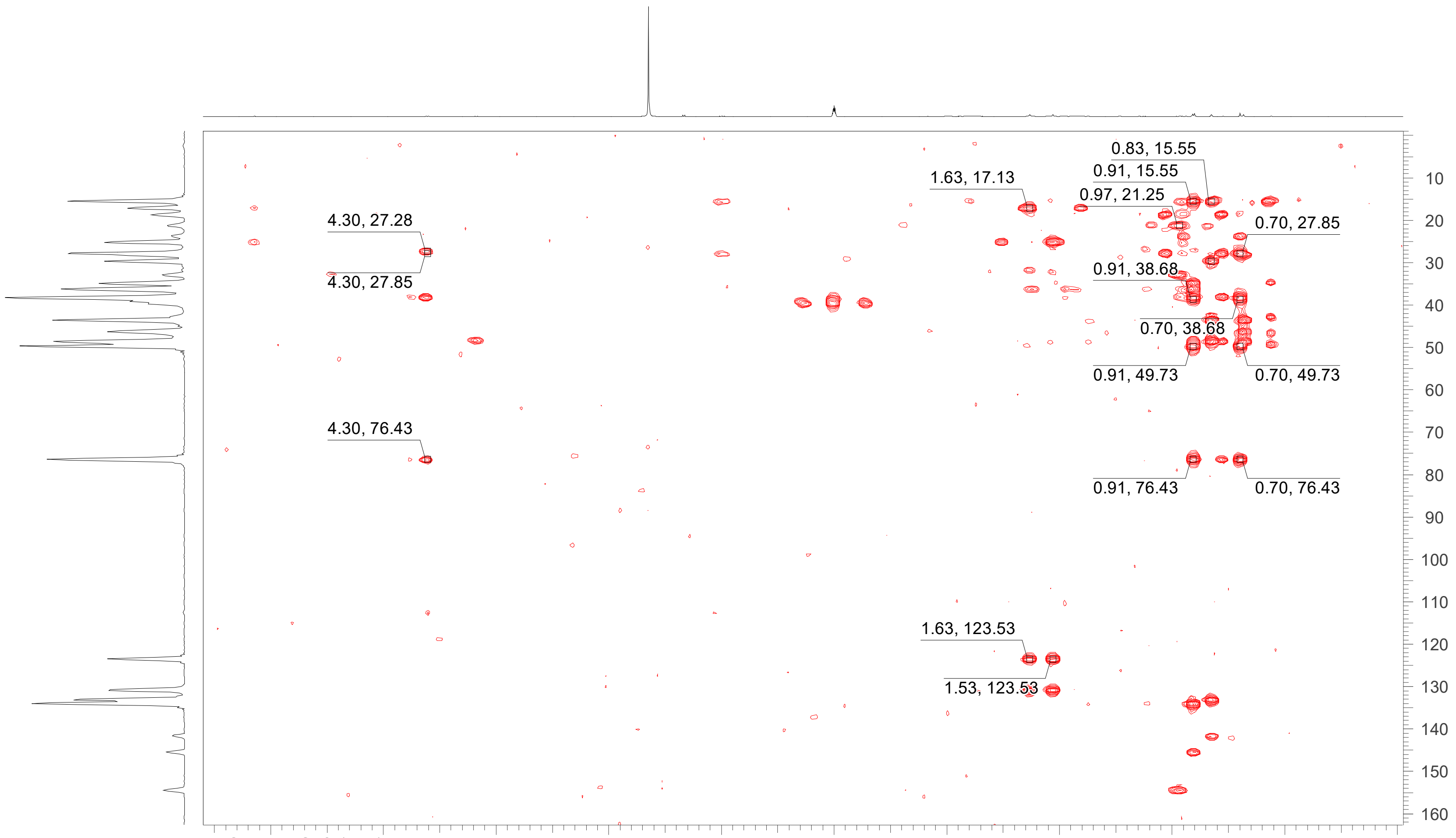

Figure S37: HMBC spectrum (DMSO-*d*₆, 700 MHz) of trametenolic acid (**6**)

Table S1: MIC assay experiment parameters

Test organisms	Strain-Nr.	Growth medium	Incubation temp. [°C]	Positive controls (references)
Schizosaccharomyces pombe	DSM70572	MYC [1]	30	Nystatin 1.0 mg/mL
Pichia anomala	DSM6766	MYC [1]	30	Nystatin 1.0 mg/mL
Mucor hiemalis	DSM2656	MYC [1]	30	Nystatin 1.0 mg/mL
Candida albicans	DSM1665	MYC [1]	30	Nystatin 1.0 mg/mL
Rhodotorula glutinis	DSM10134	MYC [1]	30	Nystatin 1.0 mg/mL
Acinetobacter baumanii	DSM30008	MHB [2]	30	Ciprobay 2.54 mg/mL
Escherichia coli	DSM1116	MHB [2]	37	Oxytetracyclin 1.0 mg/mL
Bacillus subtilis	DSM10	MHB [2]	30	Oxytetracyclin 1.0 mg/mL
Mycobacterium smegmatis	ATCC 700084	7H9+ADC [3]	37	Kanamycin 1.0 mg/mL
Staphylococcus aureus	DSM346	MHB [2]	37	Oxytetracyclin 1.0 mg/mL
Pseudomonas aeruginosa	PA14	MHB [2]	37	Gentamycin 1.0 mg/mL
Chromobacterium violaceum	DSM30191	MHB [2]	30	Oxytetracyclin 1.0 mg/mL

[1]MYC: 1 % w/v, bacto peptone, 1% w/v yeast extract, 2 % w/v glycerol, pH 6.3; [2]MHB: Müller-Hinton Broth (SN X927.1, Carl Roth GmbH, Karlsruhe, Germany); [3]7H9+ADC: Middlebrook 7H9 Broth Base + Middlebrook ADC Growth Supplement (SN M0678+M0553, Merck, Darmstadt, Germany);

[20]: Kemkuignou, B.M.; Treiber, L.; Zeng, H.; Schrey, H.; Schobert, R.; Stadler, M. Macrooxazoles a–d, new 2,5-disubstituted oxazole-4-carboxylic acid derivatives from the plant pathogenic fungus *Phoma macrostoma*. *Molecules* 2020, 25, 1–18, doi:10.3390/molecules25235497.

Antimicrobial assay protocol

The assay was conducted as a minimum inhibitory concentration (MIC) assay in 96-well roundbottom microtiter plates using the parameters summarized in Table S38 and as already described in [20].

Stocks of the test organisms were generated by growing the organisms overnight in 50 mL shaking flasks filled with 25 mL of the growth medium at 140 rpm (for media and temperatures see Table S38). If the organisms were well grown the next day, which was checked by occurrence of an optical density (OD)>30 of the suspension (OD600 nm for bacteria, OD548 nm for fungi and *M. smegmatis*), aliquots of these were stored in 1.5 mL reaction tubes in a freezer at −80 °C for up to 12 months. Upon use, aliquots were unthawed and the OD of the suspension measured and adjusted by diluting with the respective growth medium. OD600 nm was adjusted to 0.01 and OD548 nm to 0.1.

Subsequently, 150 µL of the adjusted suspensions were added to all wells of a 96-well microtiter plate (one test organism per plate). In row A, additional 130 µL of suspensions plus 20 µL of the test compounds (1 mg/mL) and the controls (one compound/column) were added. The test compounds were dissolved in MeOH, MeOH was used as negative control, while different positive controls (references) were used for the test organisms (see Table S38). Then, starting from row A, 150 µL of the suspension were transferred to the next row, the contents thoroughly mixed, and 150 µL transferred to the following row. The remaining 150 µL after row H were discarded. This resulted in a serial dilution of the test compounds, ranging from 66.7 µg/mL in row A to 0.52 µg/mL in row H. The microtiter plates were then incubated overnight on a microplate shaker at 800 rpm at 30 or 37 °C (see Table S38) and were visually evaluated the next day. The MIC is defined as the lowest concentration were no growth of the test organism was observed. A lower MIC thus corresponds to a higher antimicrobial activity of the test compound.

Table S2: Cytotoxicity assay experiment parameters

cell line	type	No.	growth medium
L929	mouse fibroblasts	ACC 2	DMEM [1] + 10 % FBS [2]
KB 3.1	Human endocervical adenocarcinoma (AC)	ACC 158	DMEM [1] + 10 % FBS [2]
A431	Epidermoid carcinoma cells	ACC91	DMEM [1] + 10 % FBS [2]
A549	Adenocarcinomic human alveolar basal epithelial cells	ACC107	DMEM [1] + 10 % FBS [2]
PC-3	Postrate cancer cells	ACC465	F12K [1] + 10 % FBS [2]
MFC-7	Breast cancer cells	ACC115	RPMI [1] + 10 % FBS [2]

Cytotoxicity assay protocol

The assay was conducted in 96-well flat-bottom microtiter plates using the parameters summarized in Table S39.

Cell lines were incubated at 37 °C under 10 % CO2 in Gibco™ DMEM medium (Thermo Fisher Scientific, Waltham, MA, USA) supplemented with 10 % FBS. A microtiter plate was filled with 120 µL of this suspension (50,000/mL) in each well.

Separately, another microtiter plate was filled with 100 µL of growth medium in each well. Then, 50 µL of the test compound solutions (1 mg/mL) were given to wells of the first column in two replicates (one compound per row). Cells without additives, MeOH were used as negative control. Starting from the first column, 50 µL of the solutions were gradually transferred to the next column, the contents thoroughly mixed, and 50 µL transferred to the following column. This created a serial dilution of the test compounds ranging from 333 µg/mL to 1.9×10−3 µg/mL. The remaining 50 µL after column twelve were discarded. From this microtiter plate, 60 µL of the solutions from 111 µg/mL to 1.9×10−3µg/mL were given to the first plate containing 120 µL of the cell suspensions (i.e. the highest concentration 333 µg/mL was not used). This resulted in final compound concentrations ranging from 37 µg/mL to 0.6×10−3 µg/mL.

After 5 days of incubation under the aforementioned incubation conditions, the half maximum inhibitory concentrations (IC50) were determined using a colorimetric tetrazolium dye MTT assay [S3]. For this, 20 µL of a 5 mg/mL solution of 3-(4,5-dimethyl-2-thiazolyl)-2,5-diphenyl-2H-tetrazolium bromide (MTT) were added to each well and incubated for two hours at 37 °C. Then, the microtiter plate was centrifuged (3,000 rpm, 5 min) and the supernatant removed by holding the plate upside-down and gentle shaking. Afterwards, the wells were washed using 100 µL of phosphate buffered saline (PBS). The plate was again centrifuged and the supernatant removed as described before. Then, 100 µL of an isopropanol:

HCl solution (1L isopropanol+4 mL HCl 37 % w/v) were added to the wells. After incubating for 10 min at ambient temperature, the absorption of the wells at 595 nm was measured with an Infinite® 200 Pro microplate reader (TECAN, Männedorf, Schweiz).

Table S3: Antimicrobial and cytotoxic activities of compounds **1** and **2**.

**Strain**	**Laetiporin C**	**Laetiporin D**	**Positive Control (μg/mL)**	
Bacteria	MIC (μg/mL)			
Bacillus subtilis DSM 10	-	-	4.2	Oxytetracyclin
Chromobacterium violaceum DSM 30191	-	-	0.83	Oxytetracyclin
Escherichia coli DSM 1116	-	-	1.7	Oxytetracyclin
Acinetobacte baumanii DSM 30008	-	-	0.26	Oxytetracyclin
Mycobacterium smegmatis ATCC 700084	-	-	1.7	Kanamycin
Pseudomonas aeruginosa PA14	-	-	0.21	Gentamycin
Staphylococcus aureus DSM 346	-	-	0.21	Oxytetracyclin
Fungi				
Candida albicans DSM 1665	-	-	4.2	Nystatin
Mucor hiemalis DSM 2656	66.7	-	8.3	Nystatin
Pichia anomala DSM 6766	-	-	4.2	Nystatin
Rhodoturula glutinis DSM 10134	-	-	1.0	Nystatin
Schizosaccharomyces pombe DSM 70572	-	-	4.2	Nystatin
Cell lines	Cytotoxicity IC_{50} (μg/mL)			
KB 3.1 HeLa	*	*	1.7×10^{-5}	epothilone B
L929	**	**	2.4×10^{-4}	epothilone B
A431	**	**	2.6×10^{-5}	epothilone B
PC-3	**	**	4.8×10^{-5}	epothilone B
A549	**	**	3.4×10^{-5}	epothilone B

MCF-7	**	**	1.5 × 10^{-5}	epothilone B

(-) no activity; starting concentration for antimicrobial assay and cytotoxicity assay were 66.7 and 300 µg/mL, respectively.
(**) no altered cells, only inhibition of proliferation, no cytotoxic activity
(*) no altered cells, no cytotoxic effect
